# Clustering the Brain With “CluB”: A New Toolbox for Quantitative Meta-Analysis of Neuroimaging Data

**DOI:** 10.3389/fnins.2019.01037

**Published:** 2019-10-22

**Authors:** Manuela Berlingeri, Francantonio Devoto, Francesca Gasparini, Aurora Saibene, Silvia E. Corchs, Lucia Clemente, Laura Danelli, Marcello Gallucci, Riccardo Borgoni, Nunzio Alberto Borghese, Eraldo Paulesu

**Affiliations:** ^1^DISTUM, Department of Humanistic Studies, University of Urbino Carlo Bo, Urbino, Italy; ^2^NeuroMI, Milan Centre for Neuroscience, Milan, Italy; ^3^Center of Developmental Neuropsychology, ASUR Marche, Pesaro, Italy; ^4^Psychology Department and PhD Program in Neuroscience of the School of Medicine and Surgery, University of Milano-Bicocca, Milan, Italy; ^5^fMRI Unit, IRCCS Istituto Ortopedico Galeazzi, Milan, Italy; ^6^Department of Informatics, Systems and Communication, University of Milano-Bicocca, Milan, Italy; ^7^Department of Economics, Management and Statistics, University of Milano-Bicocca, Milan, Italy; ^8^Psychology Department, University of Milano-Bicocca, Milan, Italy; ^9^Department of Computer Science, Università degli Studi di Milano, Milan, Italy

**Keywords:** coordinate-based meta-analysis, clustering, non-parametric statistics, cognitive dimensions, anatomical segregation, clusters composition

## Abstract

In this paper we describe and validate a new coordinate-based method for meta-analysis of neuroimaging data based on an optimized hierarchical clustering algorithm: CluB (Clustering the Brain). The CluB toolbox permits both to extract a set of spatially coherent clusters of activations from a database of stereotactic coordinates, and to explore each single cluster of activation for its composition according to the cognitive dimensions of interest. This last step, called “cluster composition analysis,” permits to explore neurocognitive effects by adopting a factorial-design logic and by testing the working hypotheses using either asymptotic tests, or exact tests either in a classic inference, or in a Bayesian-like context. To perform our validation study, we selected the fMRI data from 24 normal controls involved in a reading task. We run a standard random-effects second level group analysis to obtain a “Gold Standard” of reference. In a second step, the subject-specific reading effects (i.e., the linear t-contrast “reading > baseline”) were extracted to obtain a coordinates-based database that was used to run a meta-analysis using both CluB and the popular Activation Likelihood Estimation method implemented in the software GingerALE. The results of the two meta-analyses were compared against the “Gold Standard” to compute performance measures, i.e., sensitivity, specificity, and accuracy. The GingerALE method obtained a high level of accuracy (0.967) associated with a high sensitivity (0.728) and specificity (0.971). The CluB method obtained a similar level of accuracy (0.956) and specificity (0.969), notwithstanding a lower level of sensitivity (0.14) due to the lack of prior Gaussian transformation of the data. Finally, the two methods obtained a good-level of concordance (AC_1_ = 0.93). These results suggested that methods based on hierarchical clustering (and *post-hoc* statistics) and methods requiring prior Gaussian transformation of the data can be used as complementary tools, with the GingerALE method being optimal for neurofunctional mapping of pooled data according to simpler designs, and the CluB method being preferable to test more specific, and localized, neurocognitive hypotheses according to factorial designs.

## Highlights

- We report a new method for coordinate-based meta-analysis of brain imaging data.
- We describe the software implementation of the method called CluB—Clustering the Brain.
- The CluB method is based on hierarchical clustering of stereotactic coordinates.
- The CluB method allows for quantitative characterization of the cognitive dimensions.
- A formal comparison between CluB and GingerALE is performed.

## Introduction

### Quantitative Meta-Analysis of Neuroimaging Data: the Past

In the past decades there has been an explosive growth in the use of neuroimaging techniques to explore the neurofunctional correlates of cognitive functions, such as memory, language, action and motor planning, as well as the neurofunctional and neuromorphometrical changes that characterize the life-cycle of neurological and psychiatric pathologies.

Indeed, while in 2000 only 9,334 fMRI papers were published, in 2017^1^ about 19,178 appeared in the international peer-reviewed literature. This “scientific boom” produced such a huge amount of empirical evidence that sometimes it can be easy to lose one's bearings. This issue becomes even more problematic when it is necessary to translate this type of experimental findings in the clinical practice, i.e., in what has been called translational medicine. For example, more than 40 papers used the voxel-based morphometry to identify the pattern of brain atrophy typically associated with Alzheimer's disease (AD) and the most of them reported a significant decrement of the gray matter density within the hippocampal formation and the medial temporal lobe regions. This finding had become so stable in the international literature, and it has been replicated with several neuroimaging *in-vivo* and *post-mortem* investigations, that the presence of documented hippocampal volumetric reductions had been included in the revised clinical criteria for the diagnosis of probable AD since 2007 (Dubois et al., 2007; McKhann et al., 2011). This is to suggest that neuroimaging studies represent an important source of evidence not only for basic cognitive neuroscience, but also for the treatment and management of psychiatric, neurological and neuropsychological deficits. In such a translational perspective, it is self-evident that the development of rigorous statistical methods to “sum-up” large amount of data represents an important milestone for cognitive and clinical neuroscience.

In the past, let's say before the 2000, the most common approach to pool together the neuroimaging data coming from different studies was based on merging the activation foci (i.e., the stereotactic coordinates corresponding to fMRI activations) reported in several independent experiments into a table (see, for example, Démonet et al., 1996; Cabeza and Nyberg, 2000) or a summary picture (see for example, Berlingeri et al., 2008). In these cases, the evaluation of the between-studies concordance was completely subjective: the scientist that was taking the responsibility to review the literature had to decide whether a certain brain region was consistently associated with either a specific task, or a specific cognitive dimension, either on the basis of the spatial proximity between the activation peaks reported in the figure, or exploring the spatial contiguity of the anatomical labels reported in the table, without, however, applying any kind of objective data-driven statistic method. Although valuable, these studies cannot be referred to as meta-analyses due to their level of subjectivity, and now they can be rather considered like “illustrated reviews.”

To overcome these limitations, in the past years several new computerized methods designed to classify the neuroimaging data reported in the literature have been developed.

Historically, Goutte et al. (2001) were the first to use hierarchical clustering to perform meta-analyses of neuroimaging data: in their initial paper entitled “Feature-Space Clustering for fMRI Meta-Analysis” they suggested that clustering algorithms could be adopted to pull-together the fMRI results reported in the literature: “*An investigator interested in comparing methods would typically produce activation maps for the different analyses and find some kind of consensus by identifying regions that ‘activate in the same way' on the different maps. The proposed meta-clustering method is a way of automating this process”* (Goutte et al., 2001; page 170).

Hierarchical clustering has become a relatively popular meta-analytical method for some time: even though it is less used than other methods (see below) it has been the basis of some with well-cited papers (e.g., Jobard et al., 2003; Salvador et al., 2005; Shehzad et al., 2009; Liakakis et al., 2011) and a method of choice of our group (Cattinelli et al., 2013a; Crepaldi et al., 2013; Paulesu et al., 2014; Zapparoli et al., 2017; Devoto et al., 2018).

Other methods for meta-analysis have rapidly appeared in the literature: the highly popular Activation Likelihood Estimates (ALE) technique (Turkeltaub et al., 2002, 2012; Eickhoff et al., 2009), with more that 300 papers easily identified on MEDLINE; the multilevel kernel density analysis—MKDA—method of Wager et al. (2007) or the signed differential mapping approach (Radua et al., 2012).

It is worth noting that the description of the pros and cons associated with each single method goes beyond the specific aims of this paper: excellent such reviews can be found elsewhere (see, for example, Wager et al., 2007). Recommendations on best practices when performing meta-analyses are described in Müller et al. (2018).

In this paper we propose a revival and an in depth examination of the hierarchical clustering approach of brain imaging data in its implementation with a software package that we called CluB (from Clustering the Brain). From the presentation of the method, it will become apparent the specific situations where CluB offers a more flexible approach to data that can be framed according to factorial designs. As the reader shall see, CluB also offers a region-of-interest oriented interrogation of the data that may appeal to investigators with specific anatomically constrained hypotheses.

### Clustering Algorithms for Meta-Analyses and the Problem of Non-uniqueness of the Clustering Solution

**Figure 1:**
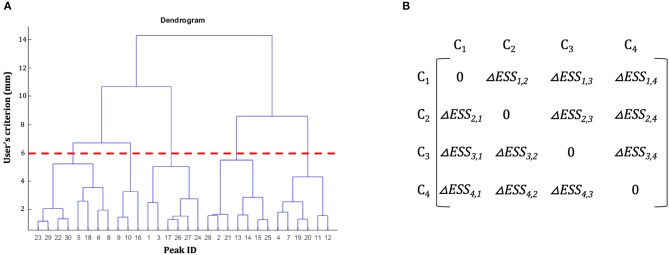
Hierarchical clustering dendrogram and dissimilarity matrix. **(A)** Example of a hierarchical clustering dendrogram. The initial dataset of peaks is represented on the x-axis. On the y-axis are represented the spatial thresholds that can be selected by the users to let the clustering solution emerge. For example, the red dotted line shows the clustering solution for a user's spatial criterion = 6 mm. **(B)** Example of a dissimilarity matrix. In each cell of the matrix is represented the difference of error sum of squares (ΔESS) between different clusters.

The basic idea underlying clustering algorithms is to group elements into subsets, called clusters, according to some homogeneity measure, so that objects inside a cluster are more similar among them, and more dissimilar from objects belonging to other clusters. In the literature several clustering methods had been developed; among these, hierarchical clustering (HC) is one of the most widely used as it has been proved to be a flexible method that can be applied to bioinformatics data (Sturn et al., 2002), medical data (Makretsov et al., 2004; Whitwell et al., 2009), and neuroimaging data (Cordes et al., 2002), for example. In what follows we briefly describe the overall logic underlying a classic HC procedure to introduce the problem of “non-uniqueness” (Cattinelli et al., 2013b). In classic HC a progressive partitioning of the data elements is achieved by iterative operations: at start, each single element represents a different cluster (partition S1) and step-by-step, two clusters are merged according to a dissimilarity measure and a new data partition is generated. The procedure is repeated iteratively to obtain a final partition containing a single cluster that includes all the clusters of the S1 partition. As a result, a hierarchy of nested clustering solutions is obtained. This solution can be represented in a tree-like structure called dendrogram (Figure 1A). The final pool of clusters, hence called the clustering solution, is obtained by cutting the dendrogram at a certain level according to what has been called “*the user's spatial criterion*” (i.e., the user decides the amount of global variability that is acceptable for the specific scientific issue of interest; in what follows we will better specify this term).

Within the clustering procedure described above, the user can play a role at different levels:

1. s/he can decide the dissimilarity measure to be adopted
2. s/he can set up the user's spatial criterion

Let's start with the first source of “subjectivity” within a clustering procedure: the choice of the dissimilarity measure. It is worthy to note that several “between-clusters dissimilarity measures” had been proposed in the literature, like for example the single linkage and Ward's method (Xu and Wunsch, 2005). In particular, the Ward's method aims at partitioning the data by minimizing the loss of information associated with each cluster. Thus, at each step the union of every possible cluster pair is considered and the two clusters whose fusion results in the minimum increase of the information loss are combined. Ward defines information loss in terms of an error sum-of-squares criterion (ESS) according to the Equation (1):

(1) $$\begin{array}{l} ESS_{k}=\;\underset{x_{i}{\varepsilon C}_{k}}{\sum }{\left(x_{i}-\;\mu_{k}\right)}^{2} \end{array}$$

Where *C*_*k*_ is one of the clusters in the partition *S* obtained at step *l*: *S* = *(C*_1_*, C*_2_*, … C*_*k*_*)*, μ_*k*_ represents the centroid of the cluster *C*_*k*_ and *x*_*i*_ is one of the element included in cluster *C*_*k*_. In general, at step *l* the error sum-of-squares for the generic cluster *C*_*k*_ (*ESS*_k_) is calculated according to Equation (1) and to obtain the minimum information loss, the measure to be minimized is

(2) $$\begin{array}{l} \Delta ESS_{k,j}=\;ESS_{k,j}-\;ESS_{k}-\;ESS_{j} \end{array}$$

Where *ESS*_*k, j*_ is the error sum-of-squares of the new cluster obtained by merging *C*_*k*_ and *C*_*j*_ at step *l* + *1*. The total cluster error sum of squares *ESS*_tot_, i.e., the total amount of variability within our partition is given by Equation (3):

(3) $$\begin{array}{l} ESS_{tot}=\;\overset{\left|C\right|}{\underset{k=1}{\sum }}ESS_{k} \end{array}$$

Where *ESS*_k_ is obtained according to Equation (1) and *|C|* is the number of clusters obtained at step *l*. The result of these operations is the creation, for each step *l* of the clustering procedure (represented by the level of the dendrogram, Figure 1A) of a dissimilarity matrix (see Figure 1B): a *|C|-by-|C| symmetric matrix* where the diagonal element is set to 0 (i.e., there is no dissimilarity between a cluster *C*_*k*_ and itself) and the remaining elements correspond to the Δ*ESS*_*k, j*_ computed according to Equation (2). Among these, in step *l* + *1* the two clusters with the lowest Δ*ESS*_*k, j*_ are merged together.

It is worth noting that this measure is computed by an iterative procedure that can suffer of a serious, although sometime neglected, problem: the final clustering solution may depend by the order of the data entered in the algorithm (van der Kloot et al., 2005) when the data to be clustered are represented by integer values, as in the case of stereotactic coordinates used in neuroimaging. This means that the same data-set arranged in a different manner could give rise to different clustering solutions due to the presence of ties in the dissimilarity matrix at a given step. In other words, the problem of non-uniqueness in the clustering solution can emerge when the minimum dissimilarity (MD) value (that is minimum value within the pool of possible dissimilarity measures between two clusters) is shared by more than one pair of clusters.

In order to overcome this problem, Cattinelli et al. (2013b) proposed a modified version of the HC that is based on the identification of critical and non-critical MD pairs. A non-critical pair consists of a pair of elements (clusters) that do not share any element with other MD pairs; thus even if they are merged together at different merging sequences, they do not result in different dendrograms. In the Catinelli's algorithm the non-critical pairs are identified and merged together in a random order producing, as a consequence, a single dendrogram that represents the scaffolding structure to build up sets of alternative dendrograms. All the possible alternative dendrograms are grouped into classes of equivalent clustering solutions. Clustering solutions are considered equivalent if, notwithstanding the shapes of the dendrograms diverge in the intermediate step, they do converge in pooling together the same set of data (Cattinelli et al., 2013b). The iterative process ends with the creation of all the possible non-equivalent dendrograms (neD). At this stage, different solutions are generated by cutting each dendrogram according to the users' spatial criterion. For each solution “*neD*” a quality measure is computed as follows:

(4) $$\begin{array}{l} bESS_{neD}=\overset{\left|C\right|}{\underset{k=1}{\sum }}n_{k}{\left(u_{k}-\;u_{X}\right)}^{2} \end{array}$$

Where |*C*| is the number of clusters in the solution *neD*, *n*_*k*_ is the number of elements included in cluster *C*_*k*_, *u*_*k*_ is the mean of cluster *C*_*k*_ (i.e., the centroid) and *u*_*X*_ is the grand mean of the data-set *X*. The above formula can be expressed also as:

(5) $$\begin{array}{l} bESS_{neD\;}=ESS_{tot}-\;ESS_{k} \end{array}$$

Where *ESS*_*tot*_ is the total error sum of squares (3) and *ESS*_*k*_ is the within-clusters error sum-of-squares computed at (1).

Among the *neD* solutions, the best one is the solution that maximizes the *bESS*_*neD*_; as the maximization of this measure favors a better spatial separation between clusters. This is the clustering method that is implemented as a collection of MATLAB 2016b (MATLAB and Statistics Toolbox Release 2016b, The MathWorks, Inc., Natick, Massachusetts, United States) functions in CluB to perform coordinate-based meta-analysis of neuroimaging data. The entire algorithm and the MATLAB script associated are available at the webpage: https://osf.io/4b2pc/.

### Coordinate-Based Meta-Analysis of Neuroimaging Data: Methodological Considerations

Data pooling is critical for establishing the reproducibility and the meaningful convergence of empirical evidence about functions and the structures in the human brain across life-span and pathologies. To pursue this aim, two different classes of strategies are available: (i) creating open shared databases of raw neuroimaging data; (ii) pooling together the positive findings reported in peer-review scientific literature. While the first solution would be the optimal one and the most desirable from the scientific and methodological point of view, only few attempts have been made so far (see for example ADNI: http://www.adni-info.org/, fRMIDC: https://www.nitrc.org/projects/fmridatacenter/, Human Connectome Project: http://www.humanconnectomeproject.org/, NeuroVault: https://neurovault.org, OASIS brains: http://www.oasis-brains.org, Open Neuro: https://openneuro.org, OpenfMRI: https://openfmri.org, SchizConnect: http://schizconnect.org) and nowadays the creation of open-source databases is still far from being the norm.

Alternatively, it is possible to pool together the results of multiple data-sets by collecting the information reported in the peer-review literature and in particular, by collecting the stereotactic coordinates (x, y, z) that correspond either to significant activations, or to structural changes in specific samples. This collection of processes represents the basic steps of the “coordinate-based meta-analysis” (CBMA) methods. Although, CBMAs are now fully accepted in the international literature, some methodological considerations need to be done. The first obvious limitation is that CBMAs are unlikely to be able to reproduce exactly the results of pooled image based meta-analysis (Salimi-Khorshidi et al., 2009). This information loss is inevitably due to the relative spatial sparseness of the stereotactic coordinates reported in the different scientific reports. Moreover, one has to consider that there is not a standard rule to report the activation (or structural) peaks in a scientific paper, nor in terms of thresholds applied nor in terms of number of the peaks reported from any given cluster. Indeed, different laboratories adopt different strategies to report their results. Finally, the stereotactic coordinates that emerge from a study depend on a series of methodological choices made by the researcher and in particular, on the selected experimental paradigm, on the selected baseline, on the statistical threshold adopted and on the dimension of the kernel Gaussian filter applied during the smoothing phase. Salimi-Khorshidi et al. (2011) suggest that the adoption of a voxel-wise comparison with an *uncorrected p-value* threshold is the best methodological choice to pool together stereotactic coordinates and to obtain a meta-analytic map similar to the one that we would have obtained by directly pooling together several fMRI (or MRI) data-sets. We took this suggestion to explicitly test our new meta-analytic toolbox by borrowing the “performance analyses” typically run in clinical studies.

### Aim of the Study

In what follows we will first describe the CluB toolbox and its components, while in the second part of the paper we will report the results of a validation study for this new meta-analytic method. In particular, we followed this logic: if a CBMA algorithm performs well, than it should be capable of reproducing the effects obtained by pooling together the data in standard random effect group analysis, an approach similar to the one adopted by Salimi-Khorshidi et al. (2009) and that has been further tested and validated in a more recent methodological paper (Maumet and Nichols, 2016).

Accordingly, we selected the fMRI data from normal controls involved in words and pseudo-words reading (Danelli et al., 2017). We run standard random effects second level analysis to obtain the pattern of activations associated with words and pseudo-words reading. The results were thresholded at *p* < 0.001 uncorrected and were considered our “Gold Standard” reference. In a second step, the subject-specific reading effects (namely the first-level linear contrast “reading > baseline”) were extracted at *p* < 0.001 uncorrected and all the activation peaks reported in the SPM table were saved to create a coordinates-based database. Accordingly, each single participant was treated as an independent fMRI study on reading. The coordinates-based database was used to run a meta-analysis using the CluB software and the last version of the GingerALE algorithm (version 2.3.6; Eickhoff et al., 2009). The two meta-analytic results were compared against the Gold Standard to compute performance measures, i.e., sensitivity, specificity and accuracy. We choose to compare our software with GingerALE for two simple reasons: (1) GingerALE is the most commonly used software for coordinate based meta-analysis (CBMA), (2) GingerALE and CluB are based on completely different assumptions. In fact, while GingerALE is based on the idea that each focus of activation is better represented by a probability distribution and is modeled accordingly, in CluB each activation peak is considered as a single data-point and it is not weighted or modeled any further. This means that if CluB will perform similarly to GingerALE and the two methods will reach a good level of concordance, then we will have a further measure of concurrent validity for the CluB toolbox.

## CluB—a Detailed Description of the Software

**Figure 2:**
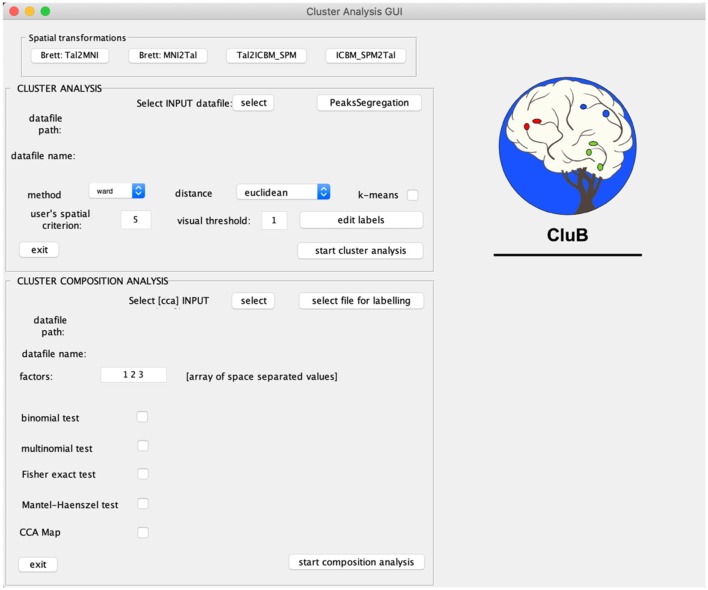
Graphical User Interface (GUI) of the CluB toolbox. Refer to section Introduction of the main text for a more detailed description of the toolbox.

CluB is designed as a collection of MATLAB functions that can be easily run through a Graphic User Interface (GUI; Figure 2). The GUI is divided into three sections that correspond to the main modules of the software: (a) Spatial transformation, (b) Cluster Analysis, and (c) Cluster Composition Analysis. Each module can be independently run and the entire procedure can be performed in separated MATLAB sessions. The module-specific results are saved in dedicated folders with specific file extensions. In what follows we will briefly describe each single module and the ensuing functions.

The **Spatial Transformation Module** permits to perform the MNI2TAL and the TAL2MNI conversion of the stereotactic coordinates included in the data-input file according to the main algorithms available in the literature (Brett et al., 2002; matthew.brett@mrc-cbu.cam.ac.uk). This preliminary step is necessary to ensure that all the activation peaks (i.e., the stereotactic coordinates) entered in the clustering procedure belong to the same anatomical space. The input file for this procedure is a tab-separated text file (.txt). The data must be entered as a matrix with N rows (as many rows equal to the number of stereotactic coordinates that have to be transformed) and three columns (i.e., the x, y, z distances from the origin of the stereotactic space). As a result, in the working directory, i.e., in the same directory of the input file, a .txt file including the N transformed peaks is saved. This file can be used to refine the entire data-set and obtain a data input made of peaks that are all conforming to the same stereotactic space for the clustering analysis.

**Figure 3:**
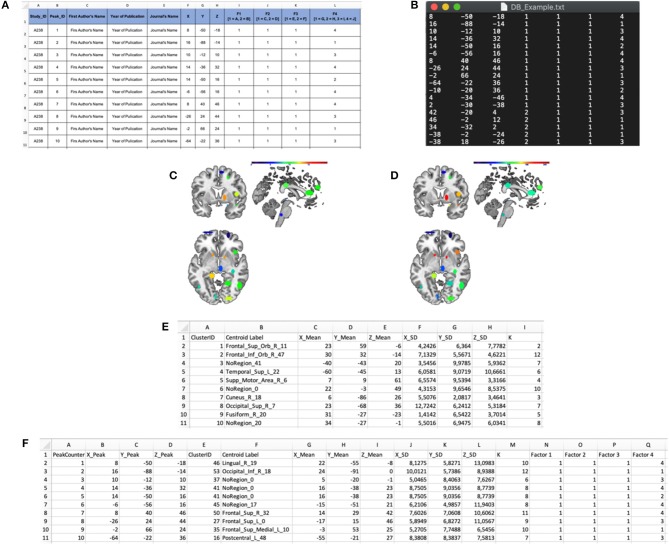
Input and output files of the “Cluster Analysis” module. **(A)** Example of a dataset in a spreadsheet file. **(B)** Example of an input .txt file. The first three columns represent the x, y, and z coordinates in stereotaxic space, whereas the last four columns represent the categorical factors (F1, F2, F3, and F4). In particular, the number in these columns represents the level of each factor for every peak. **(C)** “Cardinality” map representing the number of peaks included in each cluster. **(D)** “Density” map representing the ratio of the cardinality and the spatial extent of the cluster. **(E)** “Cluster Mapping” spreadsheet file in which the anatomical and spatial information about the clusters is stored. **(F)** “Peaks Clustering” spreadsheet file in which each peak is associated with its cluster.

**Figure 4:**
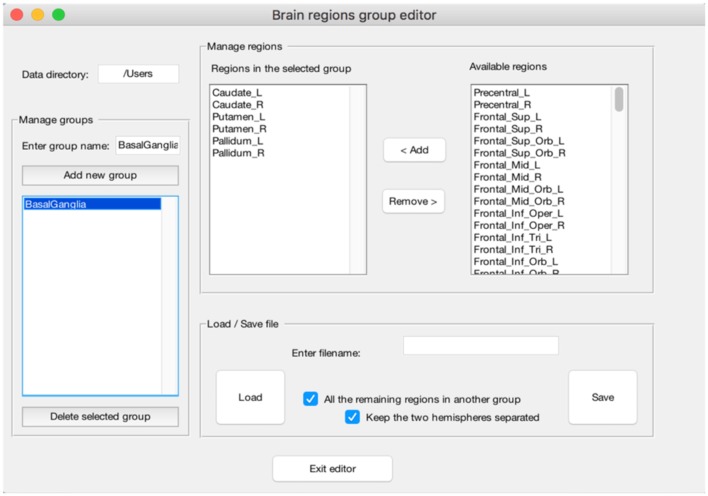
Example of the “Peaks Segregation” function. The CluB's “Peaks Segregation” module permits to perform a clustering and statistical inference on pre-defined regions or groups of regions taken from the Automatic Anatomical Labeling template (Rorden and Brett, 2000). In the figure, a new group of brain regions named “BasalGanglia” has been created (left—“Menage groups”). The list of brain regions included in the group is reported in the middle of the figure (mid-left—“Regions in the selected group”), along with the remaining brain regions (mid-right—“Available regions”). This operation will allow CluB to perform the clustering by taking into account the anatomical constraints selected by the user.

The **Clustering analysis module** is the heart of CluB. This module is based on the modified hierarchical algorithm described in Cattinelli et al. (2013b). The algorithm takes into account the squared Euclidean distance between each pair of foci included in the dataset. Then, the clusters with minimal dissimilarity are recursively merged using Ward's criterion (Ward, 1963), to minimize the intra-cluster variability and maximizing the between-cluster sum of squares (Cattinelli et al., 2013b). To run this analysis, a number of parameters need to be set:

- The **INPUT** data-file, i.e., a .txt file including the activation peaks reported in the literature together with their “cognitive” characterization (e.g., whether that particular peak was activated either in patients or in healthy subjects, and whether it was activated either in task “A” or “B”; see Figures 3A,B). The cognitive characterizations of the tasks/populations that generated the local effects represented by stereotactic coordinates are reported as numeric factor (i.e., categorical variables in which each number correspond to a level of the factor);
- The **dissimilarity measure** (at the moment only the Ward's method is implemented);
- The **user's spatial criterion**, i.e., the maximal spatial variability on average that is acceptable for the specific purpose of the study. It indicates the level at which the algorithm “cuts” the dendrogram (Figure 1A, dotted line) to obtain a set of clusters. Usually it ranges from 5 to 7.5 mm to resemble the typical spatial resolution of neuroimaging results;
- The **visual threshold**, i.e., the minimum number of activation peaks included into a cluster—e.g., if it is set to 10, then only the clusters that include at least 10 activation peaks are visualized in the digital image outputs, the clustering-maps;
- The **peak segregation tool**: this toolbox allows one to run a semantic clustering on neuroimaging meta-analysis datasets. It permits to create specific combinations of regions that will be treated separately by the clustering algorithm and the clustering solution will be constrained within the pre-defined region. This is particularly useful, for example, to avoid that cerebellar data points contribute to occipital clusters or vice-versa. A similar example can be made for the thalamus and nearby basal ganglia, or peaks nearby the mid-sagittal line of the brain in the frontal, parietal and occipital lobes. The Peaks Segregation (Figure 4) creates groups of brain regions derived from the Automatic Anatomical Labeling (AAL) template labels (Rorden and Brett, 2000). The grouping function distributes the data points in the dataset in several independent groups, as defined with the editor. The clustering function runs the Ward hierarchical clustering algorithm separately on each cluster prepared by the grouping function, obtaining one or more clusters. Then it produces a final result by putting together all the clusters obtained in each group; when necessary, it renames the cluster labels to avoid label collisions;
- The **edit labels tool**: this is used to edit the labels of the factors that are included in the INPUT data file. In particular, it is possible to attach a name at each factor and to convert each single numbered level of each factor as a string. This passage is useful to obtain a pool of output data files that can be easily read and interpreted;

The **cluster analysis module** produces the following output files:

- A cardinality clustering-map, an ANALYZE file including all the clusters above a specified visualization threshold; in this file, the intensity of the voxel values is determined by the number of peaks included in each cluster C_i_ (see Figure 3C, for an example);
  density clustering-map, a ANALYZE file including all the clusters above the visualization threshold; the intensity of the voxel values is determined by the spatial density of each cluster C_i_ (see Figure 3D, for an example);
- A ^*^ClusterMapping.xls file including the x, y, z of the centroid, the standard deviation along the three axes, the cardinality and the anatomical label of each cluster C_i_ (see Figure 3E, for an example);
- A ^*^PeaksClustering.xls file including all the activation peaks reported in the input file, the factors that characterize each activation peak, the cluster ID of each peak (see Figure 3F, for an example);
- A ^*^PeaksCompAnalysis.**cca** (the file extension cca stands for “cluster composition analysis) file containing the aforementioned information in an encrypted format (to prevent casual corruption of the input data) for statistical assessment of the significance of condition specific effects in each cluster.

The **cluster composition analysis (CCA) module**^2^ represents the most innovative part of the CluB toolbox. This module allows one to assign a “neurocognitive meaning” to each cluster by means of categorical data analyses (see Crepaldi et al., 2013 and Paulesu et al., 2014 for practical examples). The results of the tests are saved to dedicated .xls files, which are labeled according to the selected statistical test.

Once the graphical user interface (GUI) is activated, the statistical tests associated with the cluster composition analysis (CCA) can be set by using the options located in the bottom part of the GUI (Figure 1).

The CCA GUI includes six sections: (1) the select INPUT option and two text strings to visualize the path and name of the INPUT file, (2) the factors text field, (3) the *binomial test*, (4) the *multinomial test*, (5) the *Fisher's exact test for 2* × *2 interaction effects*, and (6) the *Mantel*-*Haenszel test for 2* × *2* × *2 interactions*. Of note, for each of the previous statistical tests, it is possible to save a map in ANALYZE format that displays only significant clusters, by flagging the option “CCA Map.”

The cluster composition analysis file (extension .cca) is the source one, as for any other analysis. Once the file has been loaded, the user can select one of the aforementioned tests. One important aspect left to the user's choice is the setting of the prior-likelihood; this can be specified manually or derived from the distribution in the data set for each factor.

Below we illustrate the statistical tests included in CluB with some examples. The examples were taken from analyses performed on a dataset of peaks created *ad hoc* as example, enclosed in the Supplementary Materials (ExampleForFigures.txt).

### Binomial Test

**Figure 5:**
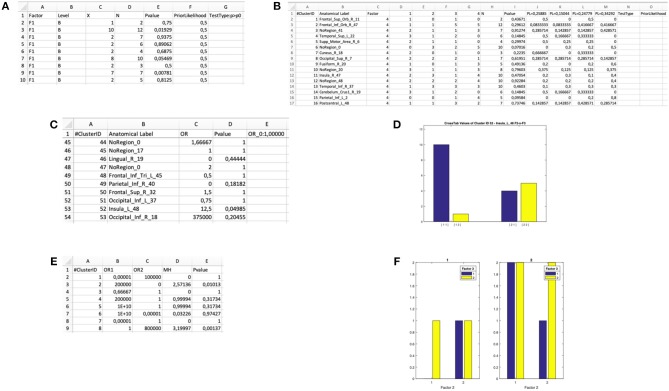
Output files of the “Cluster Composition Analysis” module and ensuing statistical inferences. **(A)** Output .xls file for a “binomial test” on the Level B of Factor F1. **(B)** Output .xls file for a “multinomial test” on Factor F4. **(C)** Output .xls file for a “Fisher's test” for 2 × 2 interaction of the Factors F2 and F3. **(D)** Bar plot generated by the “Fisher's test” procedure for 2 × 2 interactions in the Cluster Composition Analysis module. **(E)** Output .xls file for a “Mantel-Haenszel test” for 2 × 2 × 2 interaction of the Factors F1, F2, and F3. **(F)** Bar plot generated by the “Mantel-Haenszel test” for 2 × 2 × 2 interactions in the Cluster Composition Analysis module.

The software computes a binomial test within each cluster, the output is saved in a dedicated XLS file: “input file name”-binomial-“tested factor—tested level(s)”.xls (Figure 5A). The result is a matrix with as many rows as the number of clusters. For each cluster the cluster ID, the factor of interest, the category of the successful events (i.e., the level of the factor of interest), the number of observed successes, the cardinality and the *p*-value of the binomial test are displayed. The chosen null and alternative hypotheses are also printed. This would be the test of choice when one wants to evaluate the association of a cluster with one level of a two-levels factor: for example, the comparison of normal and dyslexic readers (Paulesu et al., 2014).

### Multinomial Test

The software performs a multinomial test within each cluster and saves the output in a dedicated XLS file: “input file name”-multinomial—“tested factor”.xls (Figure 5B). The result is a matrix with as many rows as the number of clusters. For each cluster, the software gives back the cluster ID, the factor of interest, the distribution of the observed frequencies (i.e., the number of peaks within level 1, level 2, and so on), the cardinality, the *p*-value. In the last *n* columns (depending on the number of levels of the factor), the prior likelihood (PL) of each level is reported, as well as the observed probability for each cluster (i.e., the proportion of foci for each level with respect to the cardinality of the cluster). This information is needed to infer which of the *n* levels significantly exceeds the PL. On the top row, the selected H0 and the selected type of multinomial test are reported. This would be the test of choice when one wants to test the association of a cluster with one level of a multi-level factor, for example, the comparison of three tasks within the same group, or the comparison of three groups.

### Fisher's Exact Test

In this case two binary factors X and Y are considered. The four possible combinations of the levels of the factors can be represented as the cells of a 2 × 2 contingency table. Also, different types of null hypothesis are considered: (1) the hypothesis of independence is *H*_0_:*OR* = 1;(2) the Prior Likelihood hypothesis is *H*_0_:*OR* = *OR*_*dataset*_ (i.e., the OR computed in the whole dataset before clustering the data). The alternative hypothesis is *H*_1_:*OR* ≠ *OR*_0_. The program computes a test in each cluster and saves the output in a XLS file: “input file name”-fisher—“tested factors”.xls (Figure 5C). The result is a matrix with as many rows as the number of clusters. For each cluster, the software returns the cluster ID, the observed odds ratio and the *p*-value. The selected null hypothesis is also printed. Finally, a bar-plot representing the observed distribution of each cluster is printed in a dedicated folder “Fisher_figures” (an example is reported in Figure 5D). This would be the test of choice, for example, when one wants to test the hypothesis that two groups of subjects differ in a given cluster specifically for one of two tasks (Paulesu et al., 2014; Devoto et al., 2018).

### The Mantel-Haenszel Test (MH)

This can be considered as an extension of the Fisher's exact test. It can be applied to explore 2 × 2 × 2 interactions by identifying the factor that, according to the specific users' hypotheses, can be considered the moderator. Similarly to the previous tests, the program computes a MH test for each cluster and saves the output in a dedicated XLS file: “input file name”—Mantel_Haenszel.xls (Figure 5E). Finally, a bar-plot representing the observed distribution of each cluster is printed in a dedicated folder “Figure-Mantel_Haenszel-tested factors” (an example is reported in Figure 5F). The joint bar-plot is split according with the factor chosen to stratify the analysis.

This test would allow one, for example, to the hypothesis that two groups (e.g., lean or overweight subjects) differ for one of two stimuli (e.g., high calorie food) providing that this is delivered during one of two physiological states (e.g., when fasting).

## Section 2: Validation Study—A Formal Comparison Between Club and Ginger ALE Based on Performance Measures

This validation focussed on the Cluster Analysis module and implied the following steps:

1. The analysis of the fMRI data of 24 subjects during a reading experiment (block-design) and the identification of a reference reading map with a standard second–level random effect analysis;
2. The extraction of individual activation peaks for the reading task from individual fixed-effects analysis;
3. The meta-analysis of the individual data collected at step 2 with both CluB and GingerALE;
4. The comparison of the meta-analyses with the reference reading map and estimates of sensitivity, specificity and accuracy for both the CluB and GingerALE analyses.

Here is worth noting that by means of this approach we treated each single subject as a single experiment in which within-study variability can be assumed to be roughly constant (as a result of the experimental task constraints), while taking mostly into account between-studies variability.

The methods of fMRI scanning are fully described in Danelli et al. (2017). The fMRI task involved 120 fMRI entire brain volumes collected in alternating blocks of 10 scans of the baseline condition and 10 scans of the experimental task (TR = 3″); thus, we had six blocks of baseline and six blocks of experimental stimuli. The participants were asked to silently read words and pseudowords. A total of 45 words and 45 pseudowords were presented in the six experimental blocks (15 for each block).

For all participants, the sampled anatomical space included the entire cerebral hemispheres and the cerebellum. For each participant, a standard pre-processing and a Hemodynamic Response Function (HRF) convolution were applied using SPM12; once obtained the smoothed-normalized-realigned-coregistered images, the two experimental conditions (baseline and reading conditions) were modeled in a first level-analysis conforming to a standard block-design. This allowed us to estimate, according to the general linear model implemented in SPM12, the subject-specific effect of interest: the contrast image (con-image) “*reading* > *baseline*” extracted at *p* < 0.001 uncorrected. The significant activation peaks were saved in an excel file to create a database for the meta-analytic procedures. As a consequence, each single subject was considered as an independent study. In particular, a total of 579 activation peaks were extracted from the 24 subject-specific *reading* > *baseline* comparison. Thus, for the 24 participants we extracted a mean of 24.2 activation peaks (min = 3; MAX = 77). The raw dataset was then passed to the GingerALE 2.3.6 software in order to exclude, from the pool of 579 activation peaks, coordinates laying outside the less conservative brain mask available within the software (this was done in order to maintain only the stereotactic coordinates located in gray matter). After this anatomical filtering process, we remained with 520 activation peaks (the 10.19% of the original dataset was eliminated). The 520 activation peaks constituted the pool of data that we used to run the two meta-analyses: one with the GingerALE method and the other with the CluB method.

The analysis with Ginger-ALE was run by setting the following parameters: (1) brain mask: less conservative; (2) uncorrected threshold *p* < 0.001; (3) no minimum cluster volume.

**Figure 6:**
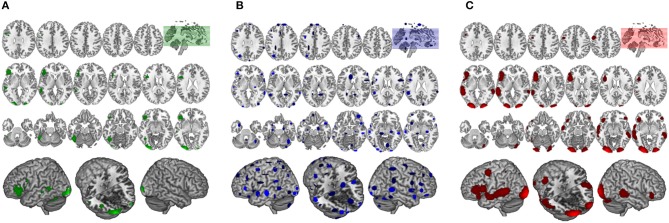
Comparison of GingerALE and CluB solutions with reference to a data set of 24 subjects involved in a reading task. Axial view (top) and 3D rendering (bottom) of the results generated by **(A)** the second-level SPM random-effects analysis (i.e., the “Gold Standard”); **(B)** the optimized hierarchical clustering algorithm implemented in CluB; **(C)** the GingerALE software.

The GingerALE method identified 10 clusters (average extended volume 8,154 mm^3^), the detailed description of the brain regions underlying reading, according to GingerALE, is reported in Table 1 and in Figure 6C.

**Table 1:** Results of the ALE analysis with a cluster forming threshold of *p* < 0.001, uncorrected.

**Cluster ID**	**Anatomical label**	**Volume (mm^**3**^)**	**Left hemisphere**			**Right hemisphere**			**Maximum ALEscore observed**
			***x***	***y***	***z***	***x***	***y***	***z***	
1	Inferior frontal gyrus, pars triangularis (47)	35,280	−42	34	0				0.0056
	Inferior frontal gyrus, pars opercularis		−48	14	10				0.0069
	Middle temporal gyrus (22)		−60	−4	−12				0.0050
			−60	−38	2				0.0066
			−62	−24	−4				0.0052
2	Inferior occipital gyrus (18)	17,520	−24	−100	−10				0.0110
3	Inferior occipital gyrus (17)	15,752				24	−102	0	0.0092
	Cerebellum					34	−80	−26	0.0045
4	Middle temporal gyrus (21)	6,272				58	−28	−8	0.0049
						62	−32	−4	0.0050
						64	−40	−2	0.0050
						64	−40	−6	0.0050
5	Precentral gyrus (6)	3,912	−48	0	54				0.0055
6	Inferior frontal gyrus, pars orbitalis (47)	1,968				46	36	−12	0.0047
7	Fusiform gyrus (37)	528	−42	−60	−20				0.0039
			−44	−48	−22				0.0040
8	Supramarginal gyrus	224	−54	−42	26				0.0039
9	Inferior parietal lobule (40)	48	−50	−46	54				0.0038
10	Middle occipital gyrus (19)	40	−28	−70	36				0.0037

*For each cluster, the volume, the coordinates in MNI stereotaxic space of the local maxima and the maximum ALEscore observed are reported*.

For the meta-analysis run with CluB the following parameters were set: (1) Users' spatial criterion: mean standard deviation along the three axes <6 mm. This was done in order to conform the spatial resolution of the CluB method to the spatial resolution applied in GingerALE 2.3.6 (the standard deviation of the Gaussian Probability Distribution used to compute the ALE maps is set to 6 mm).

The algorithm identified a total of 75 clusters scattered all over the brain, with 3–15 individual activation peaks each (median value = 6). The mean standard deviations along the three axes was 5.60 mm (x axis), 5.94 mm (y axis) and 5.78 mm (z axis). A complete list of these clusters is provided in Table 2 and the spatial distribution of the clusters (according to the cardinality and the density measures) is represented in Figure 6B^3^.

**Table 2:** Results of CluB with user's spatial criterion set to 6 mm.

	**Left hemisphere**							**Right hemisphere**						
**Anatomical label**	**μx**	**μy**	**μz**	**SDx**	**SDy**	**SDz**	***N***	**μx**	**μy**	**μz**	**SDx**	**SDy**	**SDz**	***N***
Middle frontal gyrus								39	39	40	3.06	3.06	6.93	3
Middle frontal gyrus, pars orbitalis	−36	51	−7	5.18	4.15	8.79	5	37	45	−15	8.64	8.01	4.22	10
Superior frontal gyrus	−16	19	63	6	5.03	7.02	3							
	−14	57	38	6	5.76	8	5							
Superior medial frontal gyrus								10	58	36	9.59	4.75	7.13	8
								7	38	58	8.08	1.91	4.43	4
Inferior frontal gyrus, pars orbitalis								52	34	−2	4.88	5.03	5.15	10
Inferior frontal gyrus, pars triangularis	−46	37	3	6.72	5.11	6.64	14	57	34	16	3.21	7.21	5.09	7
	−42	32	29	4.27	4.08	11.08	6							
Inferior frontal gyrus, pars opercularis	−49	14	10	2.6	4.46	2.14	8	54	9	19	13.37	4.76	6.61	4
	−45	11	24	5.01	5.89	5.99	11							
Gyrus rectus	−3	50	−19	4.16	7.21	4.16	3							
Precentral gyrus	−45	4	55	5.75	6.89	3.91	10	49	10	43	4.6	8.65	3.03	5
	−39	5	38	4.93	7.87	3.4	11	36	−10	58	2.61	8.29	10.77	5
	−27	−25	73	8.33	8.33	1.15	3							
Supplementary motor area	−4	2	68	7.21	8.41	5.92	9							
Middle cingulum	−6	22	39	3.65	8.39	6.19	4							
Postcentral gyrus	−61	−3	21	3.06	9.02	3.06	3							
	−59	−16	43	1.15	8.17	1.91	4							
Paracentral lobule								5	−27	60	8.25	3.83	6.32	4
Insula	−37	16	−4	5.93	6.84	4.77	5							
Superior parietal lobule	−31	−63	60	3.03	3.9	6.16	5							
Inferior parietal lobule	−50	−42	57	4.29	5.9	7.42	9	48	−42	56	8.29	9.63	4.97	6
Supramarginal gyrus								65	−39	26	4.76	5.51	8.49	4
Superior temporal pole	−41	28	−19	4.43	4	4.43	7	62	9	−1	2.49	9.97	5.35	8
	−28	8	−29	5.17	8.17	5.2	9	48	15	−19	7.13	5.29	8.16	12
Superior temporal gyrus	−58	4	−10	2.63	5.96	7.71	10							
	−53	−44	24	7.12	7.86	6.36	10							
Middle temporal gyrus	−62	−20	−7	4.78	6.77	8.53	15	63	−44	1	4.03	8.39	6.24	12
	−58	−36	2	3.55	2.83	2.83	7	55	−26	−12	7.17	4.45	6.81	14
	−57	−52	5	6.23	6.23	5.45	8							
Inferior temporal gyrus	−62	−41	−15	4.63	2.73	8.45	6	61	−47	−19	2.58	5.74	4.16	4
Parahippocampal gyrus	−26	−12	−24	4.87	5.18	5.18	8	18	−7	−22	5.59	8.38	4.68	7
	−14	−27	−10	9.1	5.93	6.54	5							
Hippocampus	−27	−49	14	3.46	7.02	5.26	4	25	−20	−7	7.08	5.43	9.17	9
Fusiform gyrus	−43	−63	−18	3.5	3.5	5.85	6							
	−42	−47	−24	1.98	5.35	7.29	8							
Precuneus	−10	−53	71	11.35	6.72	7.95	5	6	−51	9	9.32	5.02	8.79	5
Cuneus								7	−92	24	4.73	5.26	8.06	4
Lingual gyrus								25	−98	−13	7.48	3.99	5.64	15
								10	−76	−10	7.27	9.5	6.2	6
Superior occipital gyrus								27	−63	37	1.15	3.06	4.16	3
								20	−103	5	3.58	2.76	4.84	6
Middle occipital gyrus	−32	−73	34	6.36	7.01	9.91	10	38	−90	3	6.62	6.69	6.02	6
	−25	−100	2	5.61	4.18	4.25	12							
Inferior occipital gyrus	−43	−79	−7	7.9	5.26	4.43	4							
	−30	−93	−11	5.95	5.06	4.75	14							
	−18	−102	−11	4.86	3.24	3.66	12							
Cerebellum	−32	−80	−44	7.87	6.32	6.63	5	38	−60	−26	6.89	10.36	5.53	10
	−13	−82	−41	5.97	4.9	3.42	4	30	−76	−45	9.59	4.54	4.13	8
								29	−83	−25	5.13	4.43	4.43	7
								8	−68	−45	4.62	1	6	4
								2	−54	−31	5.51	2.83	7.72	4
Thalamus	−3	−13	8	1.91	4.12	5.97	4							
Putamen								30	5	−6	11.14	1.15	5.29	3
Pallidum	−12	3	−7	2	8.08	4.16	3							
No region	−25	−39	38	4.16	12.22	5.29	3							
	−11	23	16	9	12.56	2.34	6							

*For each cluster, the mean centroid coordinates in MNI stereotaxic space, the standard deviation along the three axes and the cardinality (N) are reported*.

Finally, the 24 con-images representing the single-subject voxel-by-voxel difference between reading and baseline were entered in a random effect second level analysis to obtain our gold-standard reference activation map. The General Linear Model (GLM) was designed to model a one-sample *t*-test and to extract the mean neural network associated with single word silent reading (once the effect of the early visual processes was eliminated). The results were extracted at *p* < 0.001, no spatial extent threshold has been adopted here. The results are reported in Figure 6A and in Table 3, and are in line with the large amount of literature available on this topic (Turkeltaub et al., 2002).

**Table 3:** Results of the random-effects second-level SPM analysis with a significance threshold set to *p* < 0.001, uncorrected.

**Anatomical label (BA)**	**Left hemisphere**					**Right hemisphere**				
	***x***	***y***	***z***	***k***	***Z*-score**	***x***	***y***	***z***	***k***	***Z*-score**
Inferior frontal gyrus, pars triangularis (47)	−44	34	0	1,198	4.19					
	−50	34	−8		3.89					
	−42	34	−10		3.89					
Pre-central gyrus (6)	−44	2	34	37	3.41					
Middle temporal gyrus (21)	−62	−50	6	279	3.83					
	−62	−30	0		3.79					
	−56	−44	8		3.63					
Fusiform gyrus (37)	−44	−56	−20	585	4.87					
	−44	−46	−26		4.16					
Inferior occipital gyrus (18)	−26	−98	−10	533	5.75	24	−100	−4	159	4.75

*For each local maxima the coordinates in MNI stereotaxic space, the cluster extent (k, number of voxels) and the Z-score are reported*.

As described at point 4, we compared the results of the two meta-analytic procedures with the results of the standard random effect analysis according to the following steps:

1. We extracted the t-map corresponding to the reference reading map (our gold-standard) from the SPM12 analyses,
2. We converted the results of our meta-analyses from .nii to .voi,
3. The .nii and the .voi files were overlapped to the “ch2bet” template available in MRIcron (Rorden and Brett, 2000) to identify, for each single meta-analytic map the brain regions shared with the reference-reading map,
4. We saved the shared regions in dedicated .voi files called GingerALE-intersection and CluB-intersection, respectively.

**Figure 7:**
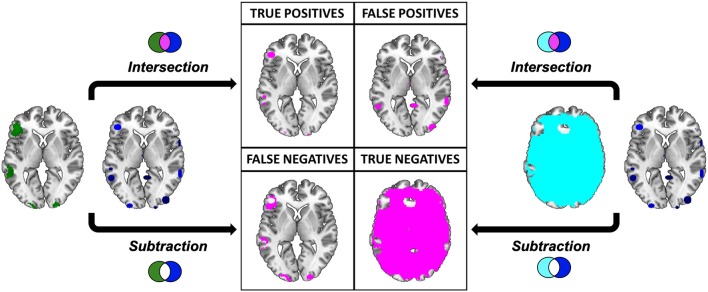
Pictorial representation of the procedure implied in the calculation of the performance measures. Graphical representation of the comparison between the results of the “Gold Standard” (green) and the meta-analytic map (CluB, in blue). Inactive voxels (i.e., voxels not displaying a significant effect in the second-level SPM fMRI results—the “Gold Standard”) are represented in cyan. The output of the comparison (i.e., intersection or subtraction) between the maps is represented in purple. The same procedure was applied to compare the meta-analytic map generated by the GingerALE software with the “Gold Standard”.

The intersections were overlaid to the “ch2bet” template and explored using the “descriptive” function available in MRIcron. As a result we obtained the anatomical distribution of the overlays and the associated voxel-count and volumetry. This result represents, for each single meta-analytic procedure, the so called true positives (TP), i.e., the voxels that are actually activated by our subjects, and that resulted to be active according to the specific pooling method of each meta-analytic algorithm (Figure 7).

To identify the true negatives (TN), i.e., the brain regions that were not active in our sample and that did not result activated in the meta-analytic procedures, we selected the mask file of the SPM 12 one sample *t*-test (i.e., the neurofunctional space mapped by our experiment) and we subtracted the reference reading map to obtain the so-called “inactive map.” Secondly, we overlapped the inactive map with each single meta-analytic map and we applied the masking procedure (i.e., a subtraction) to obtain the distribution of the TN voxels (Figure 7).

The false positives (FP, i.e., the voxels that resulted to be activated in the meta-analytic map, but that were not active in our gold-standard result) were identified by overlapping the inactive map with the results of each single meta-analysis (Figure 7). Finally, the false negatives (FN, i.e., the voxels that were significantly activated in the gold-standard map, but not detected by the meta-analysis) were obtained by subtracting the results of the meta-analysis from the reference reading map (Figure 7). As a result, we obtained the 2 × 2 contingency tables reported in Tables 4, 5, respectively. These were used to compute performance measures for the two meta-analytic procedures: sensitivity, specificity and overall accuracy.

**Table 4:** Contingency table of the second-level SPM results (i.e., the Gold-Standard) and the GingerALE map.

**GingerALE**	**Gold standard**		**Total**
	**Active**	**Inactive**	
Active	16,136	39,550	55,686
	1.16%	2.84%	4.01%
Inactive	6,087	1,328,491	1,334,578
	0.44%	95.56%	95.99%
Total	22,223	1,368,041	1,390,264
	1.60%	98.40%	100%

**Table 5:** Contingency table of the second-level SPM results (i.e., the Gold-Standard) and the CluB map.

**CluB**	**Gold standard**		**Total**
	**Active**	**Inactive**	
Active	3,083	41,872	44,955
	0.22%	3.01%	3.23%
Inactive	19,140	1,326,169	1,345,309
	1.38%	95.39%	96.77%
Total	22,223	1,368,041	1,390,264
	1.60%	98.40%	100%

In particular, sensitivity expresses the proportion of actual positives findings that are correctly identified. Thus, it represents the true positive rate [TP/(TP + FN)]. Specificity corresponds to the proportion of negatives that are correctly identified. Thus, specificity expresses the proportion of “*real*” negative findings [TN/(TN + FP)]. Finally, accuracy is calculated as the proportion of correct assessments (both positive and negative) over the entire sample [(TN + TP)/(TN + TP + FN + FP)].

The performance measures described above, and the corresponding confidence intervals (95%) were computed using the “epi.tests” function available in the “epiR” library of R (version 0.9-48, Stevenson et al., 2013).

The GingerALE method obtained the following performance scores: (1) Sensitivity = 0.728 [0.722–0.734]; (2) Specificity = 0.971 [0.97–0.971]; (3) Accuracy = 0.967 [0.966–0.967]. The CluB method obtained (1) Sensitivity = 0.139 [0.134–0.143], (2) Specificity = 0.969 [0.969–0.97], (3) Accuracy = 0.956 [0.955–0.956].

To conclude this empirical assessment, the concordance between the two meta-analytic methods was evaluated. The two meta-analytic methods were treated as “two independent classifiers.” Thus, in order to obtain a global measure of concordance between the two meta-analytic methods, a 2 × 2 contingency table was created and, as a consequence, four classes of events were considered:

1. Active voxels both in the GingerALE and in the CluB maps, that were calculated as an intersection between the two neurofunctional maps (Table 6);
2. Active voxels in the CluB map only, i.e., the result of the subtraction between the CluB map and the GingerALE map (Table 6);
3. Active voxels in the GingerALE map only, i.e., the result of the subtraction between the GingerALE map and the CluB map (Table 6);
4. Inactive voxels both in the GingerALE and in the CluB maps, that were calculated as a difference between the total number of voxels investigated and the number of voxels classified according to the previous classes of events (Table 6).

**Table 6:** Contingency table of the GingerALE and the CluB maps.

	**Ginger ALE**		**Total**
**CluB**	**Active**	**Inactive**	
Active	*(a)* 7,255	*(b)* 37,700	*(B+)* 44,955
	*0.52%*	*2.71%*	*3.23%*
Inactive	*(c)* 48,431	*(d)* 1,296,878	*(B−)* 1,345,309
	*3.48%*	*93.28%*	*96.77%*
Total	(A+) 55,686	(A−) 1,334,578	(n) 1,390,264
	4.01%	95.99%	100%

This classification was performed by computing nine clustering maps varying the user's spatial criterion from 6 to 14 mm (with steps of 1 mm)^4^. In order to overcome some of the methodological limitations encountered with the classical Cohen's Kappa measure (it has been demonstrated that the Cohen's kappa is sensitive to trait prevalence and marginal probabilities), the AC_1_ measure proposed by Gwet (2002) was adopted here. With respect to table, the AC_1_ is calculated as follows:

(6) $$\begin{array}{l} AC_{1}=\;\frac{P_{\alpha }-\;P_{e}\left(\gamma \right)}{1-\;P_{e}\left(\gamma \right)} \end{array}$$

(7) $$\begin{array}{l} P_{\alpha }=\frac{a+d}{n} \end{array}$$

(8) $$\begin{array}{l} P_{e}\left(\gamma \right)=\;2\;P_{+}\;\left(1-\;P_{+}\right) \end{array}$$

(9) $$\begin{array}{l} P_{+}=\left(\frac{A_{+}+B_{+}}{2}\right)/n \end{array}$$

Where *P*_α_ represents the observed concordance [see Equation (7)], *P*_*e*_(γ), represents the modified chance correction [see Equation (8)]; while *A*_+_*and B*_+_ represent the marginal frequency of the contingency table represented in Table 6.

**Figure 8:**
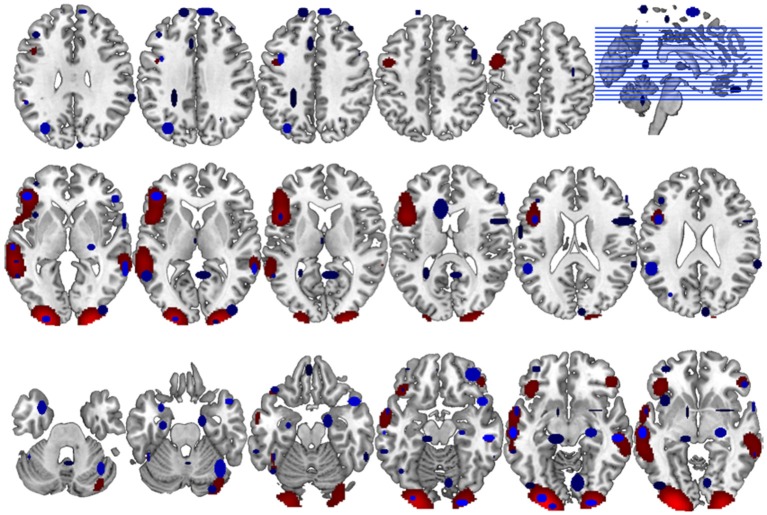
Comparison between CluB and GingerALE. Axial view of the neuroanatomical distribution of the overlap between the CluB map (in blue) and the GingerALE map (in red). These overlaps were used to compute the concordance measures between the two meta-analytic methods, as described in the main text.

Accordingly, the between methods concordance when the user's spatial criterion was set to 6 mm is AC_1_ = 0.933. The neuroanatomical distribution of the overlap between the GingerALE and the CluB maps is reported in Figure 8; among the 75 clusters identified by the CluB method, 47 (i.e., the 62.67%) fell outside the GingerALE map. The details about the concordance measures computed for the remaining clustering solutions are reported in Table 7.

**Table 7:** Concordance measures between the GingerALE map and the results of CluB with the User's Spatial Criterion set from 7 to 14 mm.

**User's spatial criterion**	**AC_**1**_**
6 mm	0.933
7 mm	0.928
8 mm	0.958
9 mm	0.942
10 mm	0.905
11 mm	0.904
12 mm	0.892
13 mm	0.897
14 mm	0.893

## Discussion

In the past decade an increasing, perhaps overwhelming, number of neuroimaging papers has been published on peer-review journals. This large amount of data represents a rich source of empirical evidence that can be used to test specific cognitive models, to explore disease-related neurofunctional and neuromorphometrical changes and to map, for example, developmental trajectories in brain functions and structures. To make the most from this source of data, a reasonable solution is to pool them by means of meta-analytic procedures. Moreover, by means of meta-analysis we can overcome, or at least minimize, the well-known methodological limitations of neuroimaging studies, such as: (1) the specific influence of the selected experimental paradigm that makes the results of a single study not necessarily bound to gain a general validity (with the well-known problem associated with the subtraction logic; Logothetis, 2008); (2) the problem of multiple comparisons and of the balance between false positive and false negative rates (Lieberman and Cunningham, 2009); (3) the typical small sample sizes of neuroimaging studies (Murphy and Garavan, 2004).

To circumvent these problems, two different classes of strategies are available: (i) to create open shared databases of raw neuroimaging data (something that is currently in the neuroimaging research agenda); (ii) to pool together the positive findings reported in peer-review scientific literature. This last option is better known as coordinate based meta-analysis (CBMA).

Although CBMAs are now fully accepted in the international literature, some methodological consideration needs to be made. As we anticipated in the introduction, the first limitation is that CBMAs are not able to reproduce exactly the results of pooled image based meta-analyses (Salimi-Khorshidi et al., 2009).

This information loss is inevitably due to the relative spatial sparseness of the stereotactic coordinates reported in the different scientific reports. Moreover, one has to consider that there is not a standard rule to report regional effects and their peaks in an imaging paper. Different laboratories adopt different strategies to report their results; some researchers can report just the local maxima for each cluster of activation, while other may decide to report more than one stereotactic coordinate for each cluster. Finally, the stereotactic coordinates that emerge from a study depend from a series of methodological choices made by the researcher and in particular, by the choice of the statistical threshold as well as by the dimension of the kernel Gaussian filter adopted during the smoothing phase. Salimi-Khorshidi et al. (2011) suggest that the adoption of a voxel-wise comparison with an *uncorrected p-value* threshold is the better methodological choice to pool together stereotactic coordinates and to obtain a meta-analytic map similar to the one that we would have obtained by directly pooling together several fMRI (or MRI) data-sets.

### CluB vs. GingerALE: Two Sides of the Same Coin

The selection of an instrument/test, as well as the development of a new tool, requires the assessment of its performance and of its accuracy. Here we adopted the measures typically used in clinical studies to assess the performance of our new toolbox, with respect to the most popular toolbox for meta-analysis of neuroimaging data: GingerALE.

From the results of our analyses a clear difference between the two methodologies emerged. The GingerALE method obtained a high level of accuracy (0.967) associated with a high sensitivity (0.728) and specificity (0.971). The CluB method obtained a similar level of accuracy (0.956), even though in the presence of imbalanced data this overall measure has to be taken “*cum grano salis”*; this was associated with similar level of specificity (0.969), notwithstanding the low-level of sensitivity (0.14).

This first result suggests that, while the two meta-analytic methods are equally accurate in identifying the true negatives, the CluB method is not as sensitive as the GingerALE method in identifying the true positive. Among the 22,223 active voxels in the *expected results* map, CluB correctly identified only 3,083 voxels (i.e., the 13.8%). However, with this regard a technical consideration needs to be made. The GingerALE method is based on the application of a three-dimensional Gaussian filter to each single activation peak collected in the input data-set. The final result is a continuously distributed anatomo-functional map (similar to the one that we would obtain with standard analyses; see the “gold-standard” map for an example). On the contrary, the CluB method creates a discrete and spatially sparse map of clusters, as one of the intrinsic aim of the hierarchical clustering procedure is to obtain a map of distinct entities whose spatial extension is limited by the choice of the “*user's spatial criterion*.” This is to say that with the GingerALE methods we are modeling the input data to simulate a standard activation map, while with the CluB method we are working on raw data (the stereotactic coordinates reported in the peer-reviewed selected literature) and we are focusing our attention to small and *ad-hoc* separated portion of the brain.

In other words, while the final result of GingerALE is more similar to an activation map devoided of information on local maxima, the final result of CluB is more similar to the summary table typically produced by a software like SPM.

While this may seem a drawback of the CluB method, from the neuroanatomical point of view, it represents one of its strengths. Indeed, the relative finer-grained and not smoothed spatial resolution of the clustering maps, together with the maintenance of the original distribution of the data, permits to explore neurofunctional segregation with a robust statistical approach (the one described in the CCA) based on non-parametric, exact tests. This is something that can be hardly done with GingerALE, which, at the most, can identify the commonalities and the differences between two sets of data (see, for example, Fornara et al., 2017), but no higher order effects like 2 × 2 interactions in a data-driven manner, starting from meta-analyses containing more than two classes of data (Paulesu et al., 2014; Devoto et al., 2018)^5^. Such higher order effects are frequently what matters the most in neuroscientific investigations.

**Figure 9:**
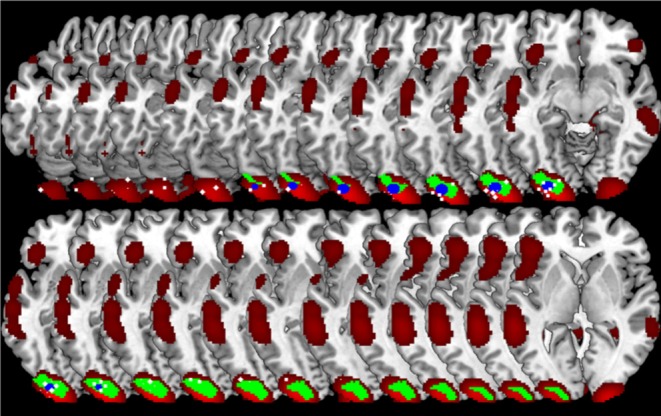
Region of interest oriented comparison of CLuB and GingerALE. The region of interest (in green, the “Gold Standard”) was taken from the SPM data analysis on 24 readers. The peaks composing the left inferior occipital cluster (*X* = −30, *Y* = −93, *Z* = −11) in the HC map (user's spatial criterion = 6 mm) are shown as 2 × 2 × 2 mm white cubic voxels. The cluster obtained in the HC map (user's spatial criterion = 6 mm) is depicted in blue. The ALE map obtained by GingerALE is depicted in red. It can be seen that while the clustering solution (in blue) is contained within the SPM “Gold Standard” map, GingerALE somewhat overestimated the activation effect as a consequence of the Gaussianization of the raw data implied. From slice in top left: *Z* = −20 to *Z* = +3 in the right bottom corner.

One final consideration needs to be made on the spatial distribution of the GingerALE map: the smoothing applied to the input data tends, somehow, to inflate the spatial distribution of the native data beyond the expected anatomical boundaries (see Figure 9 for an illustration). On the contrary CluB allows one to test specific anatomically constrained hypotheses by imposing anatomical masks on the analyses allowing one to keep separated the inference on regions that are spatially close but functionally distant or hardly equivalent; for example, the cerebellum and the occipital lobe; the thalamus and the basal ganglia. This possibility is offered by the **peak segregation module** of CluB.

As a last step of our comparative assessment, we evaluated the between-methods concordance. In particular, to overcome the problem associated with the unknown prevalence of the phenomenon investigated, we adopted the AC_1_ measure proposed by Gwet (2002). Using this measure, we found that the two meta-analytic methods have a high level of agreement (93.3%). This result is confirmed by the neuroanatomical distribution of the neuroimaging findings (see Figure 8 for more details).

To conclude, we wish to emphasize that the seemingly contrasting meta-analytic approaches compared here are in fact complementary in nature: one is based on the idea that each focus of activation is better represented by a probability distribution rather than in terms of a single data-point (i.e., the GingerALE method); CluB takes a different stance, as each activation peak is considered as a single data-point and it is not weighted or modeled any further. In other words, these two methods could be thought as two sides of the same coin, with the GingerALE method being optimal for neurofunctional mapping of pooled data, and the CluB method being the optimal choice if one wants to test more specific neurocognitive hypotheses. However, notwithstanding these two different basic ideas, from the neurofunctional point of view they reach a good level of concordance.

Finally, for those interested in a hierarchical clustering meta-analysis, the congruence between the HC solutions and those of an ALE map, corrected for multiple comparison, may permit to decide on which set of clusters to proceed with further assessments of the data using a CCA (see, for example, Paulesu et al., 2014) with the trust that the cluster considered is “spatially significant”.

### Future Directions

While creating a new toolbox, many issues may rise, and many others can emerge when reviewing the existing instruments. Among these, the first issue that we want to report as a stimulus for further development is the fact that the meta-analytic approaches considered in this paper just rely on the reported activation peaks in the peer-review literature and just consider the spatial distribution of these peaks without taking into account the magnitude of the effect of interest. Usually, the magnitude of the effect is expressed as either a *t-value* or as a *Z-score*. Thus, it may be desirable to develop new algorithms capable of taking into account also one of these measures (arguably the Z-scores as they do not depend on the degrees of freedom of the test) to obtain a better fitting of the data to the expected results. Moreover, by introducing the *t-value* or the *Z-score* associated with each activation peak, the lack of between-studies uniformity in reporting the coordinates may be reduced, as usually the papers that report the higher number of activation peaks are also those that adopted a less conservative statistic threshold (David et al., 2013). This problem has been recently addressed in the GingerALE algorithm by introducing also the study as one of the level in the analysis and hence taking into account also within-study variability. By doing this, the probability that one study with many foci drives a meta-analytic result has been mitigated (Turkeltaub et al., 2012). A similar approach has been now incorporated in the Multikernel Density Approach (MKDA).

On the contrary, at the moment this issue remains unaddressed by the CluB method. One can only recommend, as a form of best practice, to reduce the amount of data per study, when these are exceedingly redundant, by taking only the local maximum for each region of by calculating a preliminary high-resolution CluB clustering solution on the redundant study. Admittedly, this approach requires some a-priori decision on what would be a redundant style of data description.

Another important issue is the one related with the problem of inactive areas. As imaging papers only report positive findings in the form of stereotactic coordinates, the inactive voxels are just represented, both in GingerALE and CluB, by zeros. As a consequence, measurements of non-active areas are lost and this, in turn, makes impossible to evaluate whether the outcome in that particular brain region would have become significant by pooling data from different studies. This means that CBMAs methods, in general, cannot aggregate power across studies, unless the effect of every single voxel is taken into account (Costafreda, 2009).

The last methodological consideration is about the adoption of hierarchical clustering and of the Ward's method. This clustering procedure maximizes the between-cluster difference and minimizes the within cluster variability; this procedure, in turn, creates localized blobs, such as those represented in Figure 6B. The result is the emergence of localized sets of activations that, however, do not seem to fully represent the complexity of brain functioning and connection. It may be the case that the adoption of a different clustering procedure, capable of representing distributed clusters rather than localized blobs, may turn into a more adequate model of the neurofunctional effect of interest. A possible candidate for this problem may be the adoption of minimum spanning tree (Jain et al., 1999), a clustering method that is typically used, and had been fully developed, to design networks, such as computer or electrical networks (Graham and Hell, 1985). Recently, this clustering method has been adopted to identify the neural network underlying specific cognitive function in the context of functional connectivity studies (Baumgartner et al., 2001; Firat et al., 2013). The authors started from the simple assumption that brain regions that show the same type of activation may constitute spatially sparse brain networks (Carpenter and Just, 1999). Therefore, in this case the similarity measure would not be based on spatial proximity, but rather on temporal co-occurrence of brain activity. Further studies are needed to better address this issue and to develop this possibility.

## Data Availability Statement

All datasets generated for this study are included in the manuscript/Supplementary Files.

## Ethics Statement

Comitato Etico Scientifico dell'A.O. Niguarda Cà Granda, Protocol Number: 296_092013, Date of approval: 16.09.2013.

## Author Contributions

MB and EP conceived the CluB toolbox. FG, AS, and SC wrote the code of the toolbox. MB, LC, MG, and RB conceived the statistical tests in the cluster composition analysis module. NB and EP conceived and implemented the optimal clustering solution algorithm and conceived the anatomical segregation module, MB and NB implemented it. MB, FD, LD, and EP conceived and performed the validation study. MB, FD, and EP wrote the manuscript. All the authors have read and approved the final version of the manuscript.

### Conflict of Interest

The authors declare that the research was conducted in the absence of any commercial or financial relationships that could be construed as a potential conflict of interest.

## Footnotes

^1^The result has been extracted from searching in the PUBMED database the following terms: (((fMRI) AND human being) AND (“2017” [Date—Publication]: “3000” [Date—Publication])).

^2^The Cluster Composition Analysis (CCA) was not used in the validation experiment described in this paper.

^3^We did not impose a threshold of a minimum # of peaks per cluster in our validation analyses to maximize the chance of overlap between the HC solution and the uncorrected GingerALE map (*p* < 0.001 uncorrected). However, in daily practice one should avoid considering clusters with limited cardinality.

^4^Detailed information about the clustering solutions computed for the different user's criteria is reported in Tables S1–S8 in the Supplementary Materials.

^5^To make a practical example, imagine a simple scenario where two different experimental tasks (e.g., word vs. face processing) and two groups of subjects (e.g., dyslexic vs. control subjects) are taken into consideration. This scenario cannot be afforded by GingerALE, whereas CluB can handle the 2 × 2 design by testing the 2 × 2 interaction (e.g., task × group) with the appropriate test (Fisher's exact test).
