# Supplementary material for: Clustering the Brain With “CluB”: A New Toolbox for Quantitative Meta-Analysis of Neuroimaging Data
Source: Front Neurosci. 2019 Oct 22;13:1037. doi: 10.3389/fnins.2019.01037 (PMC6817507; doi:10.3389/fnins.2019.01037)
Supplement: Supplementary file 1 [file Data_Sheet_1.PDF]

Table S1 | Results of CluB with User's Spatial Criterion set to 7 mm. For each cluster, the mean centroid coordinates in MNI stereotaxic space, the standard deviation along the three axes and the cardinality (N) are reported.

	Left Hemisphere							Right Hemisphere						
	µx	µy	µz	SDx	SDy	SDz	N	µx	µy	µz	SDx	SDy	SDz	N
Inferior Frontal Gyrus, pars Orbitalis	-40	23	-13	5.31	8.16	8.50	12	52	34	-2	4.88	5.03	5.15	10
Inferior Frontal Gyrus, pars Triangularis	-42	32	29	4.27	4.08	11.08	6	57	34	16	3.21	7.21	5.09	7
	-46	37	3	6.72	5.11	6.64	14							
Inferior Frontal Gyrus, pars Opercularis	-47	12	18	4.63	5.47	8.65	19							
Rolandic Operculum								60	9	6	8.32	8.33	11.06	12
Middle Frontal Gyrus								39	39	40	3.06	3.06	6.93	3
Middle Frontal Gyrus, pars Orbitalis	-36	51	-7	5.18	4.15	8.79	5	37	45	-15	8.64	8.01	4.22	10
Superior Frontal Gyrus	-14	57	38	6.00	5.76	8.00	5							
Superior Medial Frontal Gyrus								10	58	36	9.59	4.75	7.13	8
								7	38	58	8.08	1.91	4.43	4
Gyrus Rectus	-3	50	-19	4.16	7.21	4.16	3							
Anterior Cingulum	-9	22	25	7.55	10.56	12.12	10							
Supplementary Motor Area	-7	7	67	8.59	10.70	6.24	12							
Precentral Gyrus	-45	4	55	5.75	6.89	3.91	10	36	-10	58	2.61	8.29	10.77	5
	-39	5	38	4.93	7.87	3.40	11	49	10	43	4.60	8.65	3.03	5
	-27	-25	73	8.33	8.33	1.15	3							
Postcentral Gyrus	-60	-10	33	2.31	10.55	11.53	7							
Paracentral Lobule								5	-27	60	8.25	3.83	6.32	4
Superior Parietal Lobule	-31	-63	60	3.03	3.90	6.16	5							
Inferior Parietal Lobule	-50	-42	57	4.29	5.90	7.42	9	48	-42	56	8.29	9.63	4.97	6
Supramarginal Gyrus								65	-39	26	4.76	5.51	8.49	4
Superior Temporal Gyrus	-53	-44	24	7.12	7.86	6.36	10							
	-58	4	-10	2.63	5.96	7.71	10							
Superior Temporal Pole	-28	8	-29	5.17	8.17	5.20	9	48	15	-19	7.13	5.29	8.16	12

Table S1 | Results of CluB with User's Spatial Criterion set to 7 mm. For each cluster, the mean centroid coordinates in MNI stereotaxic space, the standard deviation along the three axes and the cardinality (N) are reported.

Middle Temporal Gyrus	-57	-45	4	4.98	9.55	4.55	15	55	-26	-12	7.17	4.45	6.81	14
	-62	-20	-7	4.78	6.77	8.53	15	62	-45	-4	3.71	7.69	10.58	16
Inferior Temporal Gyrus	-62	-41	-15	4.63	2.73	8.45	6							
Fusiform Gyrus	-42	-47	-24	1.98	5.35	7.29	8							
Precuneus	-10	-53	71	11.35	6.72	7.95	5	6	-51	9	9.32	5.02	8.79	5
Cuneus								7	-92	24	4.73	5.26	8.06	4
Lingual Gyrus								25	-98	-13	7.48	3.99	5.64	15
								10	-76	-10	7.27	9.50	6.20	6
Superior Occipital Gyrus								20	-103	5	3.58	2.76	4.84	6
								27	-63	37	1.15	3.06	4.16	3
Middle Occipital Gyrus	-25	-100	2	5.61	4.18	4.25	12	38	-90	3	6.62	6.69	6.02	6
	-32	-73	34	6.36	7.01	9.91	10							
Inferior Occipital Gyrus	-18	-102	-11	4.86	3.24	3.66	12							
	-30	-93	-11	5.95	5.06	4.75	14							
	-43	-69	-14	5.25	9.10	7.93	10							
Parahippocampal Gyrus	-26	-12	-24	4.87	5.18	5.18	8							
	-14	-27	-10	9.10	5.93	6.54	5							
Hippocampus								25	-20	-7	7.08	5.43	9.17	9
								22	-4	-17	8.97	9.02	9.09	10
Vermis								5	-61	-38	5.85	7.48	10.05	8
Cerebellum, Crus I								29	-83	-25	5.13	4.43	4.43	7
								38	-60	-26	6.89	10.36	5.53	10
Cerebellum, Crus II	-23	-81	-42	12.25	5.49	5.46	9	30	-76	-45	9.59	4.54	4.13	8
Thalamus	-7	-6	1	5.38	10.09	9.30	7							

Table S1 | Results of CluB with User's Spatial Criterion set to 7 mm. For each cluster, the mean centroid coordinates in MNI stereotaxic space, the standard deviation along the three axes and the cardinality (N) are reported.

No Region	-26	-45	24	3.65	10.06	13.95	7
