# Supplementary material for: Clustering the Brain With “CluB”: A New Toolbox for Quantitative Meta-Analysis of Neuroimaging Data
Source: Front Neurosci. 2019 Oct 22;13:1037. doi: 10.3389/fnins.2019.01037 (PMC6817507; doi:10.3389/fnins.2019.01037)
Supplement: Supplementary file 2 [file Data_Sheet_2.PDF]

Table S2 | Results of CluB with User's Spatial Criterion set to 8 mm. For each cluster, the mean centroid coordinates in MNI stereotaxic space, the standard deviation along the three axes and the cardinality (N) are reported.

	Left Hemisphere							Right Hemisphere						
	**μx**	**μy**	**μz**	**SDx**	**SDy**	**SDz**	**N**	**μx**	**μy**	**μz**	**SDx**	**SDy**	**SDz**	**N**
Inferior Frontal Gyrus, pars Orbitalis	-40	23	-13	5.31	8.16	8.50	12							
Inferior Frontal Gyrus, pars Triangularis	-42	32	29	4.27	4.08	11.08	6	54	34	5	4.86	5.81	10.44	17
	-43	40	1	7.54	8.01	8.35	19							
Inferior Frontal Gyrus, pars Opercularis	-47	12	18	4.63	5.47	8.65	19							
Rolandic Operculum								60	9	6	8.32	8.33	11.06	12
Medial Frontal Gyrus, pars Orbitalis								37	45	-15	8.64	8.01	4.22	10
Medial Frontal Gyrus								46	21	42	6.39	16.80	4.66	8
Superior Frontal Gyrus								1	57	37	14.71	4.93	7.23	13
								7	38	58	8.08	1.91	4.43	4
Gyrus Rectus	-3	50	-19	4.16	7.21	4.16	3							
Anterior Cingulum	-9	22	25	7.55	10.56	12.12	10							
Supplementary Motor Area	-7	7	67	8.59	10.70	6.24	12							

Table S2 | Results of CluB with User's Spatial Criterion set to 8 mm. For each cluster, the mean centroid coordinates in MNI stereotaxic space, the standard deviation along the three axes and the cardinality (N) are reported.

Precentral Gyrus	-27	-25	73	8.33	8.33	1.15	3	36	-10	58	2.61	8.29	10.77	5
	-42	4	46	6.06	7.26	9.22	21							
Postcentral Gyrus	-60	-10	33	2.31	10.55	11.53	7							
Postcentral Lobe								5	-27	60	8.25	3.83	6.32	4
Superior Parietal Lobule	-20	-58	65	13.64	7.39	8.80	10							
Inferior Parietal Lobule	-50	-42	57	4.29	5.90	7.42	9	48	-42	56	8.29	9.63	4.97	6
Supramarginal Gyrus								65	-39	26	4.76	5.51	8.49	4
Superior Temporal Gyrus	-53	-44	24	7.12	7.86	6.36	10							
	-58	4	-10	2.63	5.96	7.71	10							
Superior Temporal Pole	-28	8	-29	5.17	8.17	5.20	9	48	15	-19	7.13	5.29	8.16	12
Medial Temporal Gyrus	-59	-43	-2	5.15	8.30	10.19	21	55	-26	-12	7.17	4.45	6.81	14
	-62	-20	-7	4.78	6.77	8.53	15	62	-45	-4	3.71	7.69	10.58	16
Precuneus								6	-51	9	9.32	5.02	8.79	5
Cuneus								7	-92	24	4.73	5.26	8.06	4

Table S2 | Results of CluB with User's Spatial Criterion set to 8 mm. For each cluster, the mean centroid coordinates in MNI stereotaxic space, the standard deviation along the three axes and the cardinality (N) are reported.

Lingual Gyrus								10	-76	-10	7.27	9.50	6.20	6
								25	-98	-13	7.48	3.99	5.64	15
Fusiform Gyrus	-42	-47	-24	1.98	5.35	7.29	8							
Superior Occipital Gyrus								27	-63	37	1.15	3.06	4.16	3
Medial Occipital Gyrus	-32	-73	34	6.36	7.01	9.91	10	29	-97	4	10.53	8.36	5.26	12
Inferior Occipital Gyrus	-25	-98	-7	7.27	5.83	7.56	38							
	-43	-69	-14	5.25	9.10	7.93	10							
Hippocampus	-21	-18	-18	8.82	9.04	9.01	13	23	-12	-12	8.08	11.17	10.44	19
Vermis								5	-61	-38	5.85	7.48	10.05	8
Cerebellum, Crus I								38	-60	-26	6.89	10.36	5.53	10
Cerebellum, Crus II	-23	-81	-42	12.25	5.49	5.46	9	29	-79	-36	7.61	5.50	11.44	15
Thalamus	-7	-6	1	5.38	10.09	9.30	7							
No Region	-26	-45	24	3.65	10.06	13.95	7							
