# Supplementary material for: Clustering the Brain With “CluB”: A New Toolbox for Quantitative Meta-Analysis of Neuroimaging Data
Source: Front Neurosci. 2019 Oct 22;13:1037. doi: 10.3389/fnins.2019.01037 (PMC6817507; doi:10.3389/fnins.2019.01037)
Supplement: Supplementary file 3 [file Data_Sheet_3.PDF]

Table S3 | Results of CluB with User's Spatial Criterion set to 9 mm. For each cluster, the mean centroid coordinates in MNI stereotaxic space, the standard deviation along the three axes and the cardinality (N) are reported.

	Left Hemisphere							Right Hemisphere						
	**µx**	**µy**	**µz**	**SDx**	**SDy**	**SDz**	**N**	**µx**	**µy**	**µz**	**SDx**	**SDy**	**SDz**	**N**
Inferior Frontal Gyrus, pars Orbitalis	-40	35	-2	4.90	1.03	6.07	6	47	20	-11	5.26	4.32	4.12	4
								46	40	-17	1.63	5.00	2.58	4
								52	34	-2	4.88	5.03	5.15	10
Inferior Frontal Gyrus, pars Triangularis	-43	32	23	5.26	5.00	5.00	4	57	34	16	3.21	7.21	5.09	7
	-50	38	7	3.92	6.50	3.69	8							
Inferior Frontal Gyrus, pars Opercularis	-45	11	24	5.01	5.89	5.99	11	43	12	17	7.07	0.00	7.07	2
	-49	14	10	2.60	4.46	2.14	8	62	17	2	1.00	2.58	4.90	4
Middle Frontal Gyrus	-37	15	57	1.41	4.24	7.07	2	39	39	40	3.06	3.06	6.93	3
	-42	32	42	2.83	2.83	5.66	2							
	-36	9	36	3.27	5.50	1.38	7							

Table S3 | Results of CluB with User's Spatial Criterion set to 9 mm. For each cluster, the mean centroid coordinates in MNI stereotaxic space, the standard deviation along the three axes and the cardinality (N) are reported.

Middle Frontal Gyrus, pars Orbitalis	-36	51	-7	5.18	4.15	8.79	5	32	49	-13	5.85	7.76	4.52	6
Superior Frontal Gyrus	-16	19	63	6.00	5.03	7.02	3	21	57	38	6.43	3.06	6.00	3
	-14	57	38	6.00	5.76	8.00	5							
Superior Medial Frontal Gyrus								4	58	34	3.16	5.83	8.05	5
								7	38	58	8.08	1.91	4.43	4
Gyrus Rectus	-3	50	-19	4.16	7.21	4.16	3							
Anterior Cingulum	-17	38	19	4.24	0.00	1.41	2							
Middle Cingulum	-6	22	39	3.65	8.39	6.19	4							
Supplementary Motor Area	-9	-3	71	4.38	6.87	3.03	5	3	9	64	3.42	4.76	6.53	4
Precentral Gyrus	-27	-25	73	8.33	8.33	1.15	3	36	-10	58	2.61	8.29	10.77	5
	-47	1	54	4.41	3.70	3.28	8	49	10	43	4.60	8.65	3.03	5

Table S3 | Results of CluB with User's Spatial Criterion set to 9 mm. For each cluster, the mean centroid coordinates in MNI stereotaxic space, the standard deviation along the three axes and the cardinality (N) are reported.

	-44	-3	42	2.31	3.83	3.42	4	65	6	20	1.41	5.66	8.49	2
Postcentral Gyrus	-45	-37	65	3.06	6.43	4.16	3							
	-59	-16	43	1.15	8.17	1.91	4							
	-61	-3	21	3.06	9.02	3.06	3							
Paracentral Lobule	5	-27	60	8.25	3.83	6.32	4							
Insula	-37	16	-4	5.93	6.84	4.77	5							
Superior Parietal Lobule	-17	-49	71	8.33	4.16	11.02	3	41	-50	57	2.31	5.29	7.02	3
	-31	-63	60	3.03	3.90	6.16	5							
Inferior Parietal Lobule	-53	-45	52	2.07	3.01	3.67	6	55	-34	54	5.77	3.46	2.00	3
Supramarginal Gyrus	-58	-44	28	3.67	9.16	3.67	6	65	-39	26	4.76	5.51	8.49	4

Table S3 | Results of CluB with User's Spatial Criterion set to 9 mm. For each cluster, the mean centroid coordinates in MNI stereotaxic space, the standard deviation along the three axes and the cardinality (N) are reported.

Superior Temporal Pole	-58	4	-10	2.63	5.96	7.71	10	43	15	-28	5.29	2.00	2.83	4
	-41	28	-19	4.43	4.00	4.43	7							
	-29	17	-30	5.00	4.43	5.16	4							
Middle Temporal Pole	56	10	-18	4.43	3.65	3.65	4							
Superior Temporal Gyrus	-45	-45	19	1.15	6.63	6.22	4	63	2	-4	3.46	8.06	4.32	4
Middle Temporal Gyrus	-57	-52	5	6.23	6.23	5.45	8	60	-53	3	3.74	4.60	3.63	5
	-58	-36	2	3.55	2.83	2.83	7	65	-38	-1	3.21	3.55	7.46	7
	-63	-22	-2	5.01	6.31	4.60	10	55	-26	-9	7.87	3.90	2.43	11
	-60	-16	-16	4.34	6.39	4.98	5							
Inferior Temporal Gyrus	-62	-41	-15	4.63	2.73	8.45	6	61	-47	-19	2.58	5.74	4.16	4
								57	-24	-24	4.62	6.93	2.00	3

Table S3 | Results of CluB with User's Spatial Criterion set to 9 mm. For each cluster, the mean centroid coordinates in MNI stereotaxic space, the standard deviation along the three axes and the cardinality (N) are reported.

Fusiform Gyrus	-43	-63	-18	3.50	3.50	5.85	6							
	-42	-47	-24	1.98	5.35	7.29	8							
Precuneus	-27	-49	14	3.46	7.02	5.26	4	1	-59	70	1.41	4.24	2.83	2
								10	-48	15	10.00	3.46	3.06	3
Cuneus								7	-92	24	4.73	5.26	8.06	4
Lingual Gyrus								16	-84	-6	3.46	3.46	6.00	3
								16	-102	-15	4.34	2.61	4.82	5
Superior Occipital Gyrus								27	-63	37	1.15	3.06	4.16	3
								20	-103	5	3.58	2.76	4.84	6
Middle Occipital Gyrus	-33	-73	39	7.47	8.46	3.80	7	38	-90	3	6.62	6.69	6.02	6
	-30	-74	22	2.00	2.00	10.00	3							

Table S3 | Results of CluB with User's Spatial Criterion set to 9 mm. For each cluster, the mean centroid coordinates in MNI stereotaxic space, the standard deviation along the three axes and the cardinality (N) are reported.

	-25	-100	2	5.61	4.18	4.25	12							
Inferior Occipital Gyrus	-43	-79	-7	7.90	5.26	4.43	4	30	-96	-11	3.75	3.05	5.82	10
	-18	-102	-11	4.86	3.24	3.66	12							
	-30	-93	-11	5.95	5.06	4.75	14							
Amygdala	-28	2	-28	5.90	1.41	5.55	5							
Parahippocampal Gyrus	-20	-25	-14	3.46	6.11	3.46	3	21	-27	-16	1.15	1.15	4.00	3
	-26	-12	-24	4.87	5.18	5.18	8	18	-7	-22	5.59	8.38	4.68	7
Vermis	-1	-55	0	1.41	4.24	2.83	2	4	-69	-14	3.46	6.11	3.46	3
								2	-54	-31	5.51	2.83	7.72	4
Cerebellum, Crus I								41	-69	-25	3.35	5.22	5.02	5
								29	-83	-25	5.13	4.43	4.43	7

Table S3 | Results of CluB with User's Spatial Criterion set to 9 mm. For each cluster, the mean centroid coordinates in MNI stereotaxic space, the standard deviation along the three axes and the cardinality (N) are reported.

Cerebellum, Crus II	-13	-82	-41	5.97	4.90	3.42	4	25	-79	-44	7.01	3.03	3.85	5
	-32	-80	-44	7.87	6.32	6.63	5	39	-71	-48	5.03	1.15	3.46	3
Cerebellum VI								34	-52	-28	8.17	6.16	6.23	5
Cerebellum VIII	8	-68	-45	4.62	1.00	6.00	4							
Thalamus	-3	-13	8	1.91	4.12	5.97	4							
Caudate	-9	15	15	9.85	5.29	1.15	4							
Putamen								27	-17	-2	7.87	3.03	7.04	6
								30	5	-6	11.14	1.15	5.29	3
Pallidum	-12	3	-7	2.00	8.08	4.16	3							
No Region	-25	-39	38	4.16	12.22	5.29	3							
	-4	-30	-3	0.00	5.66	1.41	2							
