# Supplementary material for: Clustering the Brain With “CluB”: A New Toolbox for Quantitative Meta-Analysis of Neuroimaging Data
Source: Front Neurosci. 2019 Oct 22;13:1037. doi: 10.3389/fnins.2019.01037 (PMC6817507; doi:10.3389/fnins.2019.01037)
Supplement: Supplementary file 4 [file Data_Sheet_4.PDF]

Table S4 | Results of CluB with User's Spatial Criterion set to 10 mm. For each cluster, the mean centroid coordinates in MNI stereotaxic space, the standard deviation along the three axes and the cardinality (N) are reported.

	Left Hemisphere							Right Hemisphere						
	**μx**	**μy**	**μz**	**SDx**	**SDy**	**SDz**	**n**	**μx**	**μy**	**μz**	**SDx**	**SDy**	**SDz**	**n**
Inferior Frontal Gyrus, pars Triangularis	-43	40	1	7.54	8.01	8.35	19	54	34	5	4.86	5.81	10.44	17
Inferior Frontal Gyrus, pars Opercularis	-46	17	21	4.85	9.85	10.17	25							
Anterior Cingulum	-9	22	25	7.55	10.56	12.12	10							
Superior Medial Frontal Gyrus								2	53	42	13.48	9.72	11.24	17
Middle Frontal Gyrus, pars Opercularis								28	46	-16	19.44	7.85	4.41	13
Supplementary Motor Area	-7	7	67	8.59	10.70	6.24	12							
Precentral Gyrus	-46	1	43	9.63	10.27	11.12	28	42	9	48	7.16	20.89	10.82	13
Paracentral Lobule	-9	-26	65	18.86	5.59	8.14	7							
Superior Parietal Lobe	-20	-58	65	13.64	7.39	8.80	10							
Inferior Parietal Lobule	-50	-42	57	4.29	5.90	7.42	9							
Supramarginal Gyrus	-42	-44	24	14.68	8.53	9.78	17	55	-41	44	11.08	8.06	16.50	10
Superior Temporal Pole	-35	17	-20	7.66	10.80	10.79	21	54	12	-7	9.48	7.45	15.64	24

Table S4 | Results of CluB with User's Spatial Criterion set to 10 mm. For each cluster, the mean centroid coordinates in MNI stereotaxic space, the standard deviation along the three axes and the cardinality (N) are reported.

Middle Temporal Gyrus	-59	-43	-2	5.15	8.30	10.19	21	59	-36	-8	6.53	11.64	9.79	30
	-61	-10	-8	4.38	13.39	8.19	25							
Fusiform Gyrus	-42	-59	-18	4.03	13.50	9.06	18							
Cuneus								15	-79	29	11.31	15.95	9.37	7
Lingual Gyrus								8	-65	-1	8.15	15.26	12.27	11
Middle Occipital Gyrus	-32	-73	34	6.36	7.01	9.91	10							
Inferior Occipital Gyrus	-25	-98	-7	7.27	5.83	7.56	38	27	-97	-5	8.97	6.21	10.00	27
Hippocampus								23	-12	-12	8.08	11.17	10.44	19
Vermis								5	-61	-38	5.85	7.48	10.05	8
Cerebellum, Crus I								33	-72	-32	8.31	12.04	10.49	25
Cerebellum, Crus II	-23	-81	-42	12.25	5.49	5.46	9							
Thalamus	-16	-14	-12	10.37	10.91	13.01	20							
No region	-16	-14	-12	10.37	10.91	13.01	20							
