# Supplementary material for: Clustering the Brain With “CluB”: A New Toolbox for Quantitative Meta-Analysis of Neuroimaging Data
Source: Front Neurosci. 2019 Oct 22;13:1037. doi: 10.3389/fnins.2019.01037 (PMC6817507; doi:10.3389/fnins.2019.01037)
Supplement: Supplementary file 5 [file Data_Sheet_5.PDF]

Table S5 | Results of CluB with User's Spatial Criterion set to 11 mm. For each cluster, the mean centroid coordinates in MNI stereotaxic space, the standard deviation along the three axes and the cardinality (N) are reported.

	**Left Hemisphere**							**Right Hemisphere**						
	**μx**	**μy**	**μz**	**SDx**	**SDy**	**SDz**	**N**	**μx**	**μy**	**μz**	**SDx**	**SDy**	**SDz**	**N**
Inferior Frontal Gyrus, pars Orbitalis								54	21	-2	7.82	12.89	14.78	41
Inferior Frontal Gyrus, pars Triangularis	-43	40	1	7.54	8.01	8.35	19							
Inferior Frontal Gyrus, pars Opercularis	-46	17	21	4.85	9.85	10.17	25							
Middle Frontal Gyrus, pars Orbitalis								28	46	-16	19.44	7.85	4.41	13
Superior Medial Frontal Gyrus								2	53	42	13.48	9.72	11.24	17
Supplementary Motor Area	-7	7	67	8.59	10.70	6.24	12							
Precentral Gyrus	-46	1	43	9.63	10.27	11.12	28	42	9	48	7.16	20.89	10.82	13
Inferior Parietal Lobule	-50	-42	57	4.29	5.90	7.42	9							
Supramarginal Gyrus	-42	-44	24	14.68	8.53	9.78	17	55	-41	44	11.08	8.06	16.50	10
Superior Temporal Pole	-35	17	-20	7.66	10.80	10.79	21							
Middle Temporal Gyrus	-59	-43	-2	5.15	8.30	10.19	21	59	-36	-8	6.53	11.64	9.79	30
	-61	-10	-8	4.38	13.39	8.19	25							

Table S5 | Results of CluB with User's Spatial Criterion set to 11 mm. For each cluster, the mean centroid coordinates in MNI stereotaxic space, the standard deviation along the three axes and the cardinality (N) are reported.

Fusiform Gyrus	-42	-59	-18	4.03	13.50	9.06	18							
Precuneus	-16	-45	65	16.47	17.26	8.27	17							
Middle Occipital Gyrus	-32	-73	34	6.36	7.01	9.91	10							
Inferior Occipital Gyrus	-25	-98	-7	7.27	5.83	7.56	38	27	-97	-5	8.97	6.21	10.00	27
Calcarine Sulcus								11	-70	11	9.85	16.71	18.77	18
Hippocampus								23	-12	-12	8.08	11.17	10.44	19
Cerebellum, Crus I								33	-72	-32	8.31	12.04	10.49	25
Cerebellum, 7b	-10	-71	-40	17.28	12.12	8.06	17							
Thalamus	-16	-14	-12	10.37	10.91	13.01	20							
Caudate	-9	22	25	7.55	10.56	12.12	10							
