# Supplementary material for: Clustering the Brain With “CluB”: A New Toolbox for Quantitative Meta-Analysis of Neuroimaging Data
Source: Front Neurosci. 2019 Oct 22;13:1037. doi: 10.3389/fnins.2019.01037 (PMC6817507; doi:10.3389/fnins.2019.01037)
Supplement: Supplementary file 6 [file Data_Sheet_6.pdf]

Table S6 | Results of CluB with User's Criterion set to 12 mm. For each cluster, the mean centroid coordinates in MNI stereotaxic space, the standard deviation along the three axes and the cardinality (N) are reported.

	**Left Hemisphere**							**Right Hemisphere**						
	**µx**	**µy**	**µz**	**SDx**	**SDy**	**SDz**	**N**	**µx**	**µy**	**µz**	**SDx**	**SDy**	**SDz**	**N**
Inferior Frontal Gyrus, pars Orbitalis								54	21	-2	7.82	12.89	14.78	41
Inferior Frontal Gyrus, pars Triangularis	-43	40	1	7.54	8.01	8.35	19							
Inferior Frontal Gyrus, pars Opercularis	-46	17	21	4.85	9.85	10.17	25							
Middle Frontal Gyrus, pars Orbitalis								28	46	-16	19.44	7.85	4.41	13
Superior Medial Frontal Gyrus	-2	41	35	12.80	17.95	13.90	27							
Supplementary Motor Area	-7	7	67	8.59	10.70	6.24	12							
Precentral Gyrus	-46	1	43	9.63	10.27	11.12	28	42	9	48	7.16	20.89	10.82	13
Superior Parietal Lobule	-28	-44	62	21.51	14.25	8.91	26							
Supramarginal Gyrus								55	-41	44	11.08	8.06	16.50	10

Table S6 | Results of CluB with User's Criterion set to 12 mm. For each cluster, the mean centroid coordinates in MNI stereotaxic space, the standard deviation along the three axes and the cardinality (N) are reported.

Angular Gyrus	-38	-55	28	12.97	16.09	10.75	27							
Superior Temporal Pole	-35	17	-20	7.66	10.80	10.79	21							
Middle Temporal Gyrus	-61	-10	-8	4.38	13.39	8.19	25	59	-36	-8	6.53	11.64	9.79	30
Inferior Temporal Gyrus	-51	-51	-9	9.40	13.47	12.68	39							
Inferior Occipital Gyrus	-25	-98	-7	7.27	5.83	7.56	38	27	-97	-5	8.97	6.21	10.00	27
Calarine Sulcus								11	-70	11	9.85	16.71	18.77	18
Hippocampus	-16	-14	-12	10.37	10.91	13.01	20	23	-12	-12	8.08	11.17	10.44	19
Cerebellum, Crus I								33	-72	-32	8.31	12.04	10.49	25
Cerebellum VIIb	-10	-71	-40	17.28	12.12	8.06	17							
