# Supplementary material for: Clustering the Brain With “CluB”: A New Toolbox for Quantitative Meta-Analysis of Neuroimaging Data
Source: Front Neurosci. 2019 Oct 22;13:1037. doi: 10.3389/fnins.2019.01037 (PMC6817507; doi:10.3389/fnins.2019.01037)
Supplement: Supplementary file 7 [file Data_Sheet_7.pdf]

Table S7 | Results of CluB with User's Criterion set to 13 mm. For each cluster, the mean centroid coordinates in MNI stereotaxic space, the standard deviation along the three axes and the cardinality (N) are reported.

	Left Hemisphere							Right Hemisphere						
	**μx**	**μy**	**μz**	**SDx**	**SDy**	**SDz**	**N**	**μx**	**μy**	**μz**	**SDx**	**SDy**	**SDz**	**N**
Inferior Frontal Gyrus, pars Orbtalis	-39	28	-10	8.65	15.24	13.99	40	54	21	-2	7.82	12.89	14.78	41
Middle Frontal Gyrus, pars Orbitalis								28	46	-16	19.44	7.85	4.41	13
Superior Medial Frontal Gyrus	-2	41	35	12.8	17.95	13.90	27							
Supplementary Motor Area	-7	7	67	8.59	10.70	6.24	12							
Precentral Gyrus	-46	8	33	7.69	12.91	15.34	53	42	9	48	7.16	20.89	10.82	13
Superior Parietal Lobule	-28	-44	62	21.51	14.25	8.91	26							
Supramarginal Gyrus								55	-41	44	11.08	8.05	16.51	10

Table S7 | Results of CluB with User's Criterion set to 13 mm. For each cluster, the mean centroid coordinates in MNI stereotaxic space, the standard deviation along the three axes and the cardinality (N) are reported.

Angular Gyrus	-38	-55	28	12.97	16.90	10.75	27							
Middle Temporal Gyrus	-61	-10	-8	4.38	13.39	8.19	25	59	-36	-8	6.53	11.64	9.79	30
Inferior Temporal Gyrus	-51	-51	-9	9.40	13.47	12.68	39							
Inferior Occipital Grus	-25	-98	-7	7.27	5.83	7.56	38							
Calcarine Sulcus								20	-86	1	12.17	17.54	16.02	45
Hippocampus	-16	-14	-12	10.37	10.91	13.01	20	23	-12	-12	8.08	11.17	10.44	19
Cerebelum, Crus I								33	-72	-32	8.31	12.04	10.49	25
Cerebellum VIIb	-10	-71	-40	17.28	12.12	8.06	17							
