# Supplementary material for: Clustering the Brain With “CluB”: A New Toolbox for Quantitative Meta-Analysis of Neuroimaging Data
Source: Front Neurosci. 2019 Oct 22;13:1037. doi: 10.3389/fnins.2019.01037 (PMC6817507; doi:10.3389/fnins.2019.01037)
Supplement: Supplementary file 8 [file Data_Sheet_8.PDF]

Table S8 | Results of CluB with User's Spatial Criterion set to 14 mm. For each cluster, the mean centroid coordinates in MNI stereotaxic space, the standard deviation along the three axes and the cardinality (N) are reported.

	Left Hemisphere							Right Hemisphere						
	**μx**	**μy**	**μz**	**SDx**	**SDy**	**SDz**	**N**	**μx**	**μy**	**μz**	**SDx**	**SDy**	**SDz**	**N**
Inferior Frontal Gyrus, pars Orbitalis	-39	28	-10	8.65	15.24	13.99	40	48	27	-5	15.99	15.97	14.30	54
Superior Medial Frontal Gyrus	-2	41	35	12.80	17.95	13.90	27							
Supplementary Motor Area	-7	7	67	8.59	10.70	6.24	12							
Precentral Gyrus	-46	8	33	7.69	12.91	15.34	53	42	9	48	7.16	20.89	10.82	13
Superior Parietal Lobule	-28	-44	62	21.51	14.25	8.91	26							
Supramarginal Gyrus								55	-41	44	11.08	8.06	16.51	10
Angular Gyrus	-38	-55	28	12.97	16.90	10.75	27							
Middle Temporal Gyrus	-61	-10	-8	4.39	13.39	8.19	25	59	-36	-8	6.53	11.64	9.79	30
Inferior Temporal Gyrus	-51	-51	-9	9.40	13.47	12.68	39							

Table S8 | Results of CluB with User's Spatial Criterion set to 14 mm. For each cluster, the mean centroid coordinates in MNI stereotaxic space, the standard deviation along the three axes and the cardinality (N) are reported.

Inferior Occipital Gyrus	-25	-98	-7	7.27	5.83	7.56	38							
Calcarine Sulcus								20	-86	1	12.17	17.54	16.02	45
Hippocampus	-16	-14	-12	10.37	10.91	13.01	20	23	-12	-12	8.08	11.17	10.44	19
Cerebellum. Crus I								33	-72	-32	8.31	12.04	10.49	25
Cerebellum VIIb	-10	-71	-40	17.28	12.12	8.06	17							
