# Supplementary material for: Clustering the Brain With “CluB”: A New Toolbox for Quantitative Meta-Analysis of Neuroimaging Data
Source: Front Neurosci. 2019 Oct 22;13:1037. doi: 10.3389/fnins.2019.01037 (PMC6817507; doi:10.3389/fnins.2019.01037)
Supplement: Supplementary file 10 [file Data_Sheet_10.ZIP › CluB/documentation.pdf]

Contents

1 Introduction **2**

2 The groups editor **3**

2.1 Parameters and invocation 3

2.2 How to use it 3

2.3 Output files 7

3 Subdividing the dataset **10**

3.1 Parameters and invocation 10

3.2 How to use it 10

3.3 Output files 11

4 Il clustering **13**

4.1 Parametri e avvio 13

4.2 Come si usa 13

4.3 Cosa produce 14

5 A complete example **15**

5.1 Groups preparation 15

5.2 Dataset distribution among the groups 17

5.3 Clustering of the dataset 17

Chapter 1

Introduction

This software allows you to run a semantic clustering on neuroimaging metanalysis datasets. With it you can divide the brain regions in several groups, that will be treated separately by the clustering, so that datapoints that belong to regions in one group aren't allowed to end in the same cluster with datapoints that fall in regions from another group.

It is composed by the following parts:

- a groups editor;
- a grouping function;
- a clustering function.

The groups editor lets you create groups of brain regions based on the AAL template labels. The grouping function distributes the datapoints in the dataset in several independent groups, as defined by the groups created with the editor. The clustering function runs the Ward hierarchical clustering algorithm searately on each cluster prepared by the grouping function, obtaining one or more clusters. Then composes the final result by putting together all the clusters obtained in each group; where necessary, it renames the cluster labels to avoid label collisions.

These three steps need to be run manually one after the other, as will be explained in the next chapters.

This program produces two text files: one with the labels of the clusters in which each datapoints has been put; the other with the informations about the clusters found. Two other XML files are written, one after the first step and one after the second, these too will be explained next.

Chapter 2

The groups editor

This module allows you to split the anatomical regions identified by the AAL template in several groups, where each region can belong to only one group.

The groups are saved to an XML file that will be used by the next module.

2.1 Parameters and invocation

The groups editor doesn't require any argument. To launch it, simply write `groups_editor4` at the Matlab console. A graphical interface opens and all the operativity is done through it.

2.2 How to use it

The editor interface is made of five different parts:

- the text field to enter the data directory;
- the controls to handle the groups;
- the controls to handle the regions within each group;
- the controls to save the groups to file or load a previously written file;
- the button to close the interface.

The editor is shown in Figure 2.1.

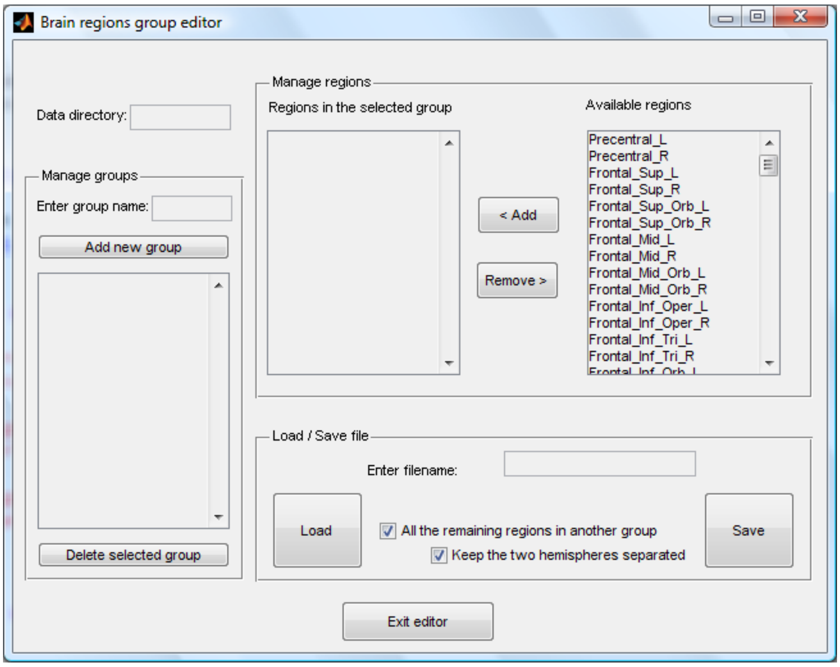

Figure 2.1: The groups editor.

2.2.1 Specifying the data folder

The text field to enter the data directory is shown in Figure 2.2.

Here we need to specify the name of the folder where the editor will save the XML file with the groups created. Entering the data directory is mandatory: the editor doesn't allow you to load or save any file if this folder hasn't been specified.

The data directory needs not to exist: in that case the editor will create it prior to save the groups.

2.2.2 The controls to manage the groups

Under the text field for the data directory, there are the controls to manage the groups, as shown in Figure 2.3.

These controls allow you create new groups: you begin by entering the name of the new group in the text field; next, by clicking on the "Add new group" button, the group is inserted in the list below. That list contains the groups you created. If you entered a group by mistake or if you at some point are no more interested in a group, you can just select it and delete it by pressing the "Delete selected group" button. That will erase the selected group from the list.

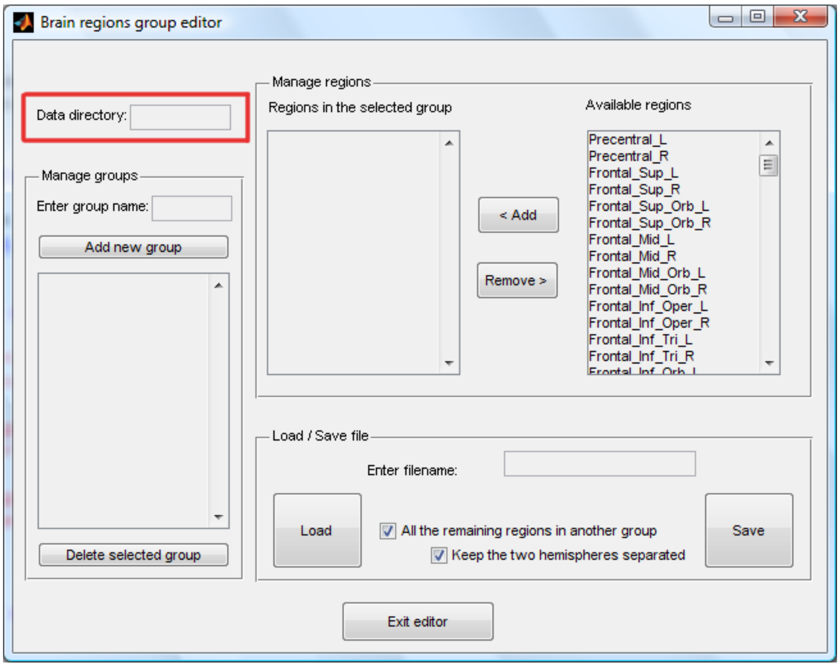

Figure 2.2: The text field to set the data directory.

2.2.3 The controls to manage the regions

On the right, at the top of the interface, there are the controls to manage the anatomical regions in each group. You can see them on Figure 2.4. On the left there's the list of regions that already are in the selected group; the list on the right contains the regions still available.

Between the two lists there are the buttons to move the regions from one list to the other. By selecting one or more entries in the "Available regions" list and by clicking on the "< Add" button, the selected regions are moved to the "Regions in the selected group" list (you need to add at least one group to be able to use this functinality). On the contrary, the "Remove >" button removes the selected regions in the list on the left and puts them back to the list on the right.

When managing the two lists, you can select one or more entries:

- by clicking on a region you select just that one;
- by keeping pressed the Ctrl key while clicking on the regions, you can select several of them one at a time;
- by selecting a region and keeping pressed the Shift key, selecting a second region all the regions between this and the first will be selected.

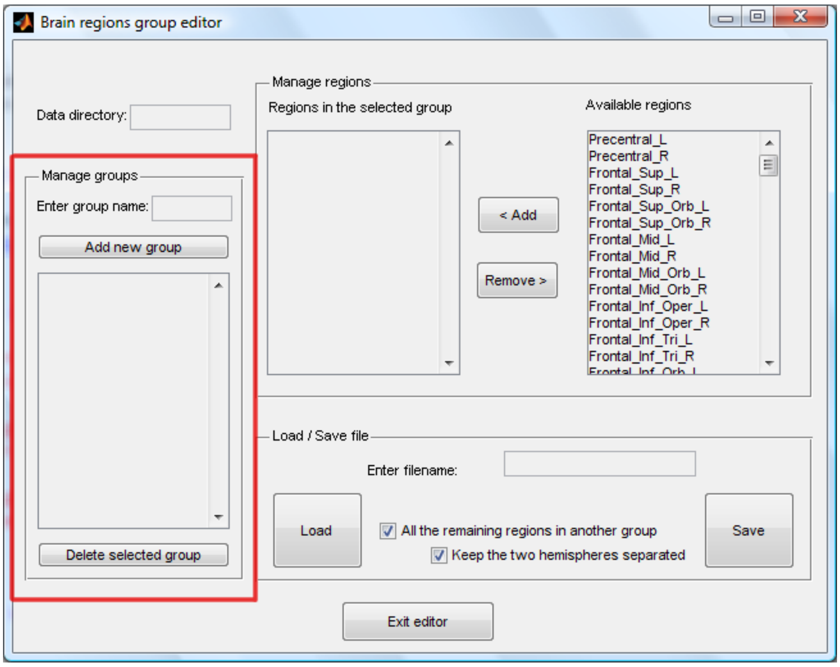

Figure 2.3: The controls to manage the groups

2.2.4 The controls to save and load files

Under the controls to manage the regions are placed those to save and load files. They're shown in Figure 2.5.

In this area you can perform two operations: save the groups you just created to a file and load a file previously written.

Let's see how to save the groups to file. In the text field you have to enter the name of the file that you want to save; before saving the groups, though, two decisions must be made regarding the regions still available (those on the list on the right in the above controls). Two checkboxes let you decide how to handle the regions that are not part of any group:

- "All the remaining regions in another group" makes the editor create an additional group in which all the regions in the list are put, all together (this group will be called *autoRemainingRegions*);

- "Keep the two hemispheres separated" makes the editor save the available regions in several groups instead of only one, there will be a group for the regions that are in the left hemisphere (it will be called *autoRemainingRegionsLeft*), a group for the regions in the right hemisphere (called *autoRemainingRegionsRight*) and a group for the regions in the cerebellar vermis, that do not belong

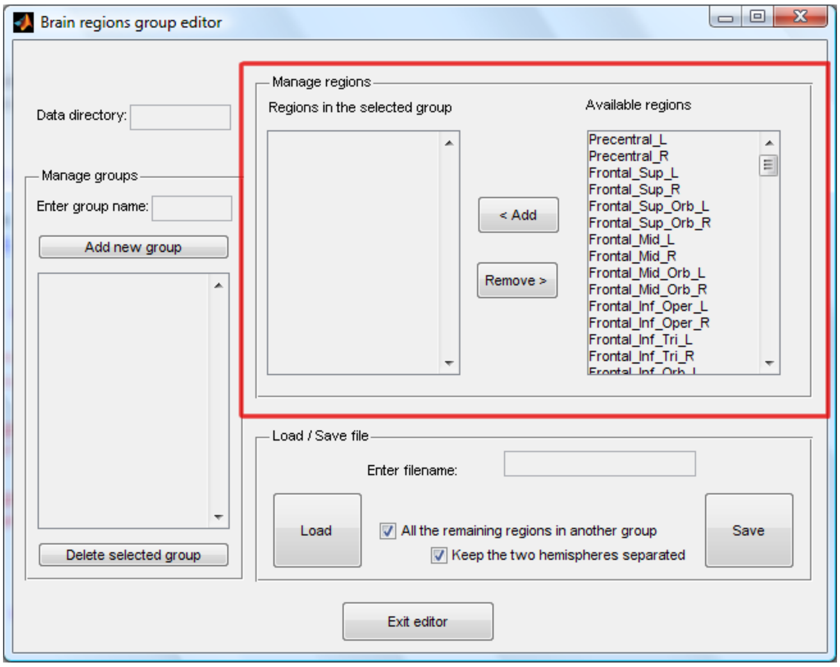

Figure 2.4: The controls to manage the regions within each group

to any cerebral hemisphere (called *autoRemainingRegionsMiddle*).

After having entered the filename (which must have the .xml extension) and having choose how the remaining regions have to be treated, by pressing the "Save" button the editor will write the specified XML file in the data directory already indicated.

You can also load an XML document with groups made previously: all you have to do is to specify the name of the file that you want to load and press the "Load" button; in this way the groups in the file will be loaded in the editor and you can eventually modify them.

2.2.5 Closing the editor

The last element of the editor is the closing button; it is placed in the lower part of the interface and can be seen in Figure 2.6. By pressing this button the editor closes and you are back to the Matlab prompt.

2.3 Output files

When you press the "Save" button, the groups editor writes an XML document, a text file that contains the groups you have defined in the interface; for each group

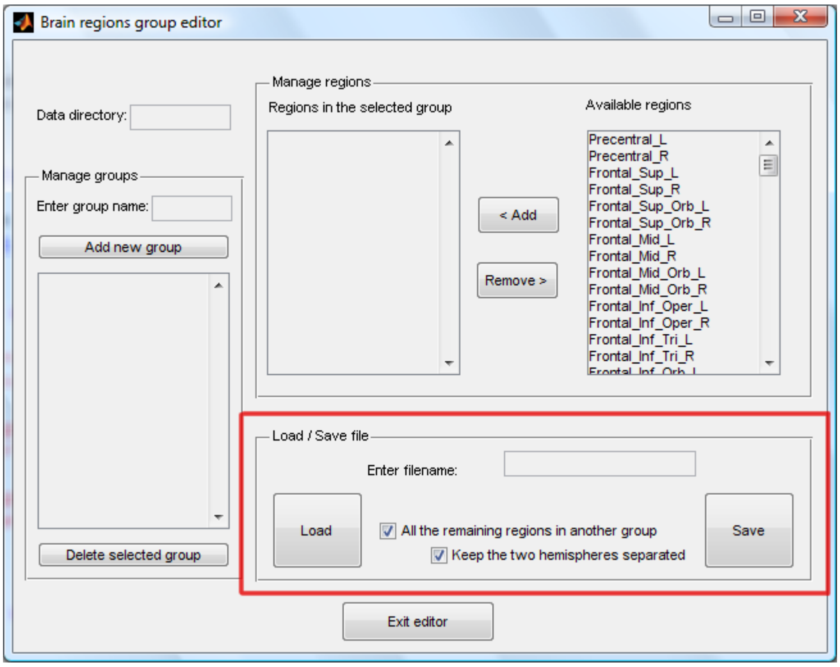

Figure 2.5: The controls to save and load files

it stores the anatomical regions that you have added to it. Its basic form is:

```
<?xml version="1.0" encoding="utf-8"?>
<grouping>
    <comment>Space for a comment</comment>
    <groups>
        <group name="group 1 name">
            <region>AAL label of the region 1</region>
            <region>AAL label of the region 2</region>
            [...]
            <region>AAL label of the region n</region>
        </group>
        <group name="group 2 name">
            <region>AAL label of the region 1</region>
            <region>AAL label of the region 2</region>
      <region>AAL label of the region 3</region>
            [...]
      <region>AAL label of the region n</region>
        </group>
        [...]
    </groups>
</grouping>
```

At the beginning of the document, you can see a node with a textual comment; this allows you to enter a short note about the file, the program will ignore this part.

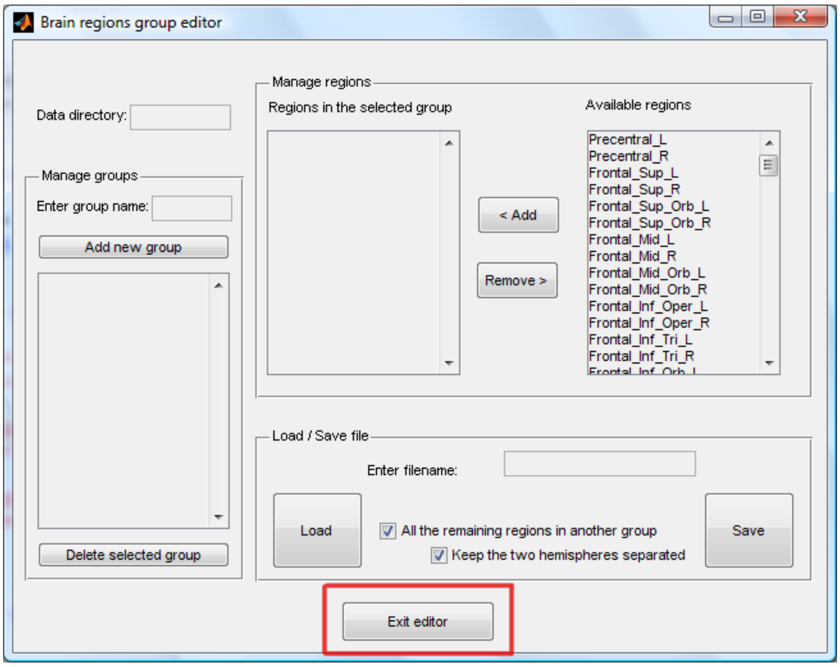

Figure 2.6: The button to close the editor

Next, there are the groups defined in the editor: each `<group>` node has an attribute that stores the group name you entered in the editor; inside this node there's a variable number of `<region>` nodes, one for each region this group contains (as in the editor, the regions are identified by their AAL labels).

The set of all regions inside each group reflects how that group has been created through the editor; all together, the groups constitute what you have produced with the interface.

The XML file is written to the data folder indicated by the text field at the top left corner of the graphical user interface. You can't save a group if you haven't specified the data directory: if you try, an error message shows up and no file is written. When the editor has finished saving the groups, a confirmation message appears.

In addition to that, although there's no visual hint about it, the editor writes a secondary file, named *init.txt*, where it saves the path to the data directory. This file will be used by all the subsequent modules to load the files and write the results.

Chapter 3

Subdividing the dataset

This module distributes the datapoints contained in the dataset you are going to analyze in several distinct groups, with each datapoint belonging to one and only one group.

3.1 Parameters and invocation

This phase is carried over by a function, named `steppedhc_grouping()`. It takes three parameters:

- *dataset*, the file with the datapoints to distribute in groups;
- *groupsxml*, the XML file with the groups made with the editor;
- *groupingxml*, the XML file where this function will save the groupings.

The first two are input parameters, the third is an output parameter.

It has to be invoked from the Matlab prompt:

» steppedhc_grouping(*dataset*, *groupsxml*, *groupingxml*);

3.2 How to use it

This function works almost automatically, it reads each datapoint in the dataset, refers to the AAL template to find the label of the region to which that datapoint belongs, and stores that datapoint in the right group for the region found.

Sometimes, the AAL template doesn't give a label for the datapoint, so the function pauses and asks you what it has to do. This is done presenting the following question:

```
The datapoint [x y z] doesn't belong to any group.

Groups available:
1: group 1 name
2: group 2 name
...
n: group n name
Specify the code of the group to which you want to add
that datapoint (ENTER to skip this, # to skip all):
```

As you can see, it first tells you the coordinates of the datapoint, next it shows you the groups that are defined in the XML document and then it asks you what it should do. Here you have two options: you can move that datapoint to one of the available groups or you can skip it and go on, in the latter case that datapoint will not take part in the clustering phase. To skip this datapoint you have to press the "#" on your keyboard and then press the ENTER key; you also have the chance to skip all the datapoints that can't be automatically assigned to the groups, just press the ENTER key alone.

At the end of the processing, the function confirms the grouping telling you where it saved the grouped datapoints:

```
XML with the grouping written to datadir/groupingxml
```

Note that it wrote the file to the data directory you entered in the editor, although you didn't put it in the function arguments. This is possible because the function reads the *init.txt* file that the editor has written when you saved the groups.

3.3 Output files

This function saves the groupings to the file you specified as third argument. This is another XML document, in which each datapoint has been assigned to a group. The XML document has the following structure:

```
<?xml version="1.0" encoding="utf-8"?>
<grouping>
    <names>
        <name groupid="-1">none</name>
        <name groupid="1">group 1 name</name>
```

```
        <name groupid="2">group 2 name</name>
        [...]
        <name groupid="n">group n name</name>
    </names>
    <peaks>
        <peak>
            <coords>datapoint 1 coordinates</coords>
            <groupid>id corresponding to the group
                     this datapoint has been put into</groupid>
        </peak>
        [...]
        <peak>
            <coords>datapoint n coordinates</coords>
            <groupid>id corresponding to the group
                     this datapoint has been put into</groupid>
        </peak>
    </peaks>
</grouping>
```

It is composed of two parts: in the first lines, the `<names>` node contains the names of the groups you created with the editor, along with the id that is used in the subsequent parts of this same document; inside the `<peaks>` node in the next part, each datapoint in the dataset is reported in a `<peak>` node, with its coordinates and the id of the group where it has been placed.

As you can see this is the same id that appears at the top of the document: that's because here you can edit the groupings: you can reassing a datapoint to another group, you can move it outside all the groups that will take part to the clustering phase (by putting it in the *none* group, writing -1 in the `<groupid>` node) and you can add a datapoint to a group by replacing -1 with the id of the new group.

If you remember, while processing the dataset the function from time to time found a datapoint with no AAL label, and prompted you about what to do with it. You had the chance to move that datapoint to an existing group or leaving it apart. All the datapoints that you didn't manually assign to a group aren't discarded, but have been assigned to the *none* group and given the id -1. By editing this XML file you have another chance to move them to a group or continue keeping them out of the clustering phase.

Chapter 4

Il clustering

Questa è la fase di clustering del dataset. I picchi distribuiti nei gruppi durante la fase precedente vengono caricati e ciascun gruppo viene clusterizzato separatamente attraverso l'algoritmo di clustering gerarchico di Ward.

Alla fine del processo i cluster ottenuti in ciascun gruppo sono riuniti nel risultato globale, e le etichette dei singoli cluster vengono rinominate dove necessario, onde evitare che due cluster abbiano la stessa etichetta.

4.1 Parametri e avvio

La funzione che sovrintente questa fase è `steppedhc_clustering()`. I suoi parametri sono:

- *groupingxml*, il file con i picchi divisi nei gruppi;
- *clusteringDataOut*, il file dove scrivere le informazioni dei cluster prodotti;
- *clusteringIdsOut*, il file su cui scrivere le etichette dei cluster.

4.2 Come si usa

La funzione va richiamata indicato il file della distribuzione dei picchi in gruppi creato nella fase precedente; in aggiunta, occorre specificare due file di testo, uno dove verranno scritte le informazioni dei cluster e l'altro dove verranno scritte le etichette dei cluster.

4.3 Cosa produce

Il clustering produce due file, i cui nomi sono stati indicati attraverso il secondo e il terzo parametro della funzione.

Nel primo trovano posto le informazioni dei cluster prodotti, un elenco di righe così costituite:

- tre valori numerici decimali, le coordinate del punto medio del cluster;
- tre valori numerici decimali, che corrispondono alla deviazione standard del cluster nelle tre direzioni;
- un valore numerico intero che indica la cardinalità del cluster.

In questo file esiste una corrispondenza biunivoca tra il numero di una riga e l'etichetta di un cluster, riportata in un secondo file.

Il secondo file scritto dalla funzione è un elenco di etichette, una per ciascuna riga, che pone in relazione il particolare dato con l'etichetta del cluster in cui è inserito.

Anche le informazioni di questo file hanno un ordine preciso: l'etichetta che si trova alla riga i corrisponde al picco i nel file del dataset.

Chapter 5

A complete example

After the deep explanation of how this editor works, and after having seen all the details of each of its parts, it's time to show you a complete example, from the creation of the groups to the results of the clustering of the dataset with this software.

To show you how it works, we'll focus on the left brain hemisphere, separating the basal ganglia and the thalamus from the rest of the brain. We further ask that the basal ganglia and the thalamus are kept separate.

5.1 Groups preparation

The first step in the procedure is to create the groups of regions. So, we start the groups editor by issuing the command `groups_editor4` at the Matlab prompt:

```
» groups_editor4
```

After the editor loads, we have to specify the name of the data directory where the groups will be saved, by writing its name in the data directory text field. Here, we enter *myexample*.

Next, we need to create two groups, one for the basal ganglia and another for the thalamus. We write *ganglia_left* in the group name text field, and then we press the "Add new group" button. This causes a group with the entered name to be added to the groups list right under that button. For the thalamus, we create the group *thalam_left* following the same procedure.

We now have two groups in the groups list on the left. This can be seen in Figure 5.1.

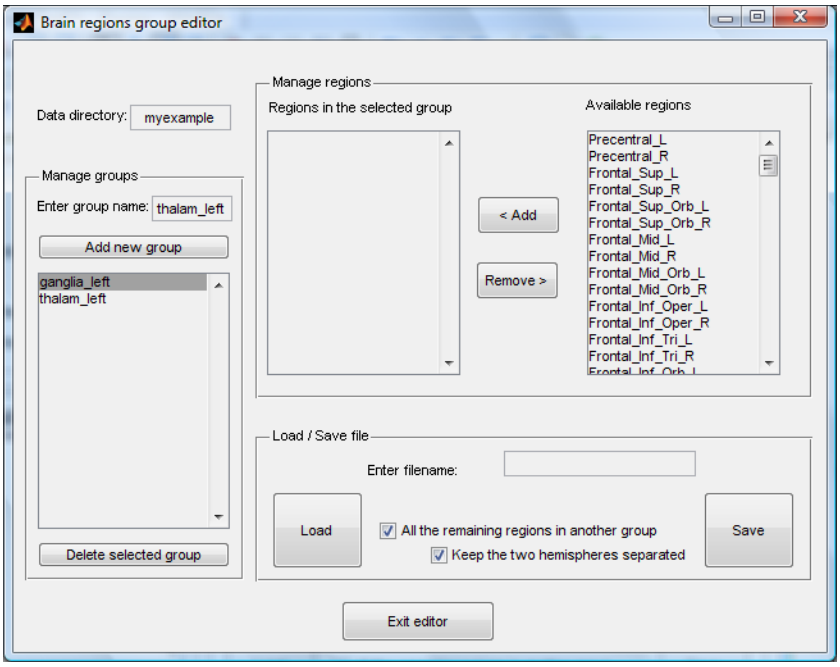

Figure 5.1: Creation of the groups.

After that, we have to add the anatomical regions to the two groups. At first, we filled the group for the basal ganglia by selecting it and, on the right part of the interface, by moving the regions *Putamen_ L*, *Caudate_ L* and *Pallidum_ L* from the ”Available regions” list to the ”Regions in the selected group” list. This is done by selecting the regions on the right list and pressing the ”< Add” button between the two lists.

The result of this operation can be seen on Figure 5.2. We also add the region *Thalamus_ L* to the *thalam_ left* group in the same way as for the other group (this isn't shown on the figure).

Finally, we need to save the groups to a file. We do this by entering the name of an XML document in the filename text field, in the botton part of the editor. We enter *example.xml*. Before saving, we ask the editor to save all the remaining regions (those in the ”Available regions” list) too, and to keep the two hemispheres separated by putting all the regions of the left hemisphere in a group and those of the right hemisphere in another one. We do this by selecting both the checkboxes beside the ”Save” button. At last, we save the groups by pressing the ”Save” button. The editor informs us that the file has been saved in our data directory with a message window, which can be seen on Figure 5.3.

5.2 Dataset distribution among the groups

The second step of the procedure involves distributing the peaks in the dataset to the groups we just created. To do this we call `steppedhc_grouping()` passing in the dataset, the XML document we made with the editor and a second XML file where the function will save the groupings generated. Here we use a dataset from a neuroimaging metanalysis on the motory imagination and we choose to save the groupings to *example_ out.xml*. Let's invoke the function from the Matlab prompt:

```
» steppedhc_grouping('motIm.txt', 'example.xml', 'example\_out.xml')
```

There's no need to specify the data directory, since we entered it in the groups editor and it took care to write its name to the configuration file *init.txt*; this function reads that configuration file to know where to look for the dataset, for the groups file and where to write the groupings file.

The function starts to read the dataset one peak at a time and looks for the group where it must go, based on the AAL label it reads from the AAL atlas. For those peaks that have no label, the procedure halts and prompts for

5.3 Clustering of the dataset

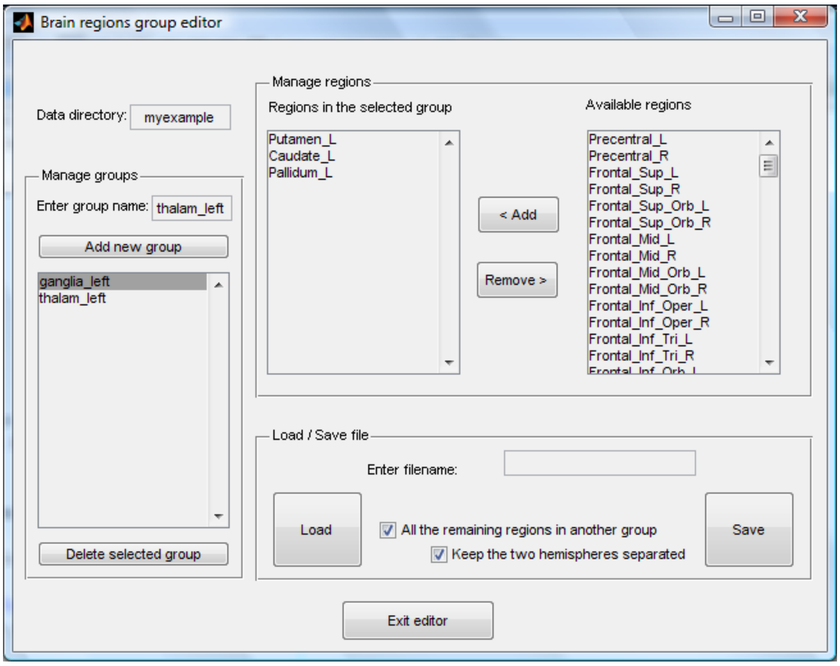

Figure 5.2: Addition of the regions to the groups.

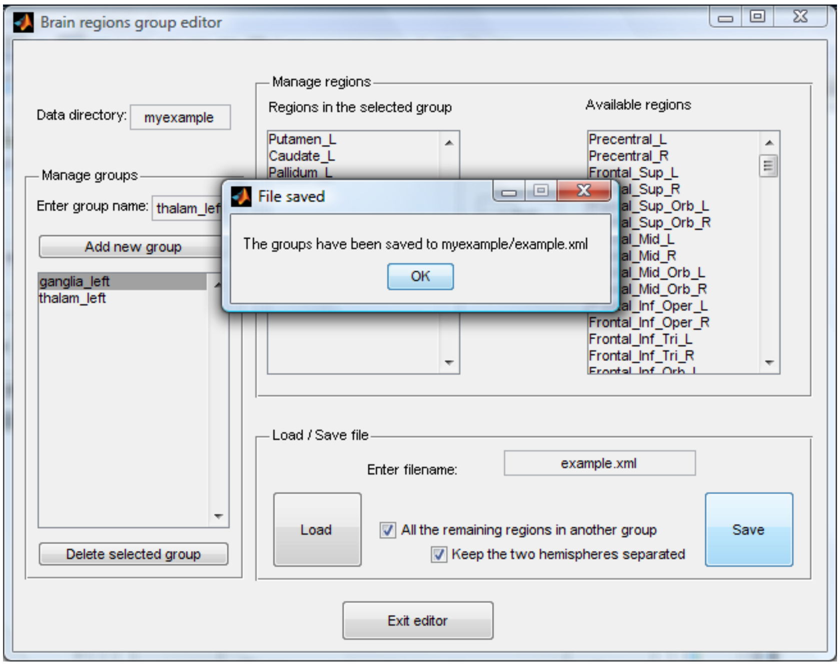

Figure 5.3: Save of the groups to file.
