# Supplementary figures and images for: Clustering the Brain With “CluB”: A New Toolbox for Quantitative Meta-Analysis of Neuroimaging Data
Source: Front Neurosci. 2019 Oct 22;13:1037. doi: 10.3389/fnins.2019.01037 (PMC6817507; doi:10.3389/fnins.2019.01037)

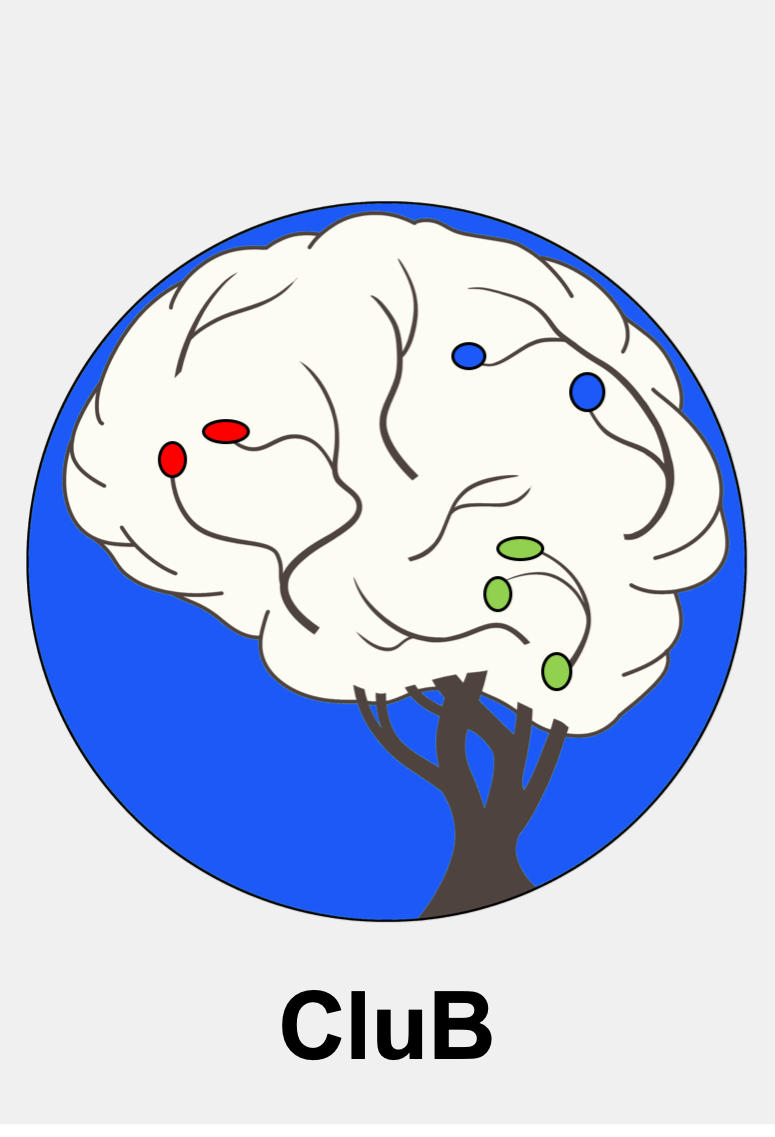

Supplement: Supplementary file 10 [file Data_Sheet_10.ZIP › CluB/guis/Clu-B.png]
